# Neurodegenerative Diseases in Children: A Comprehensive Review

**DOI:** 10.3390/ijms27094096

**Published:** 2026-05-03

**Authors:** Constantin Ailioaie, Laura Marinela Ailioaie, Cristinel Ionel Stan, Anca Sava, Dragos Andrei Chiran

**Affiliations:** 1 Department of Medical Physics, Alexandru Ioan Cuza University, 11 Carol I Boulevard, 700506 Iasi, Romania; lauraailioaie@yahoo.com; 2 Grigore T. Popa University of Medicine and Pharmacy Iasi, 16 Universitatii Street, 700115 Iasi, Romania; cristinel.stan@umfiasi.ro (C.I.S.); sava.anca@umfiasi.ro (A.S.)

**Keywords:** pediatric neurodegeneration, lysosomal storage disorders, leukodystrophies, mitochondrial diseases, peroxisomal disorders, developmental regression, neuroinflammation, oxidative stress, gene therapy, precision medicine

## Abstract

Neurodegenerative diseases (NDDs) in children represent a heterogeneous group of rare but collectively significant disorders characterized by progressive neurological decline, developmental regression, and substantial morbidity and mortality. Unlike adult-onset neurodegeneration, pediatric conditions are predominantly genetic and frequently arise from defects in fundamental cellular pathways, including lysosomal degradation, mitochondrial oxidative phosphorylation, peroxisomal lipid metabolism, and myelin maintenance. This comprehensive review synthesizes current knowledge regarding the epidemiology, molecular classification, pathophysiology, and emerging therapeutic strategies of major pediatric neurodegenerative disorders. Epidemiological data indicate a “rare-but-many” landscape, where individually uncommon diseases collectively impose a measurable population burden. Mechanistically, disease progression reflects converging processes such as toxic substrate accumulation, impaired autophagy–lysosome flux, mitochondrial bioenergetic failure, oxidative stress, neuroinflammation, and glial dysfunction. Representative groups discussed include lysosomal storage disorders, leukodystrophies, mitochondrial encephalopathies, peroxisomal disorders, and other monogenic neurodegenerative syndromes. Advances in next-generation sequencing, metabolic profiling, and neuroimaging have substantially improved diagnostic accuracy and enabled earlier detection, including through newborn screening programs. Therapeutic paradigms are shifting from primarily supportive care toward mechanism-based interventions, including enzyme replacement therapy, hematopoietic stem cell transplantation, substrate reduction strategies, and gene therapy approaches. Early molecular diagnosis is increasingly recognized as critical for optimizing outcomes, particularly in disorders amenable to presymptomatic intervention. Continued integration of genomic medicine, standardized epidemiologic surveillance, and translational research will be essential to refine disease classification, improve prognostication, and expand access to targeted therapies. Collectively, pediatric neurodegenerative diseases exemplify the intersection of developmental neurobiology and inherited metabolic dysfunction, underscoring the need for multidisciplinary, precision-based clinical strategies.

## 1. Introduction

Neurodegenerative diseases in children represent a major diagnostic, therapeutic, and social challenge due to their rarity, marked clinical heterogeneity, and frequently progressive, life-limiting course. Although individually uncommon, these disorders collectively account for a substantial burden of morbidity, disability, and premature mortality in the pediatric population. In addition to their profound effects on neurological development and quality of life, they impose considerable emotional, social, and economic strain on families and healthcare systems.

In contrast to the more prevalent neurodegenerative disorders of adulthood, such as Alzheimer’s disease or Parkinson’s disease, pediatric neurodegenerative diseases are predominantly caused by inherited genetic defects. These pathogenic variants disrupt essential cellular pathways involved in lysosomal degradation, mitochondrial energy production, peroxisomal metabolism, protein trafficking, autophagy, and myelin formation or maintenance. As a result, the developing nervous system is particularly vulnerable to progressive injury, often manifested by developmental delay, regression of previously acquired milestones, epilepsy, movement disorders, cognitive decline, and multisystem involvement. The phenotypic spectrum is broad, and substantial overlap exists among disease categories, which frequently complicates early recognition and diagnostic classification.

Over the last decade, advances in next-generation sequencing, metabolomic testing, and neuroimaging have significantly improved diagnostic precision and expanded the recognized spectrum of pediatric neurodegenerative conditions. At the same time, the therapeutic landscape has begun to evolve from predominantly supportive care toward mechanism-based interventions, including enzyme replacement therapy, hematopoietic stem cell transplantation, substrate reduction therapy, small-molecule approaches, and gene-based treatments for selected disorders. These developments have increased the importance of timely diagnosis, particularly in conditions in which early or presymptomatic intervention may modify disease trajectory.

This review is based on several key assumptions. First, pediatric neurodegenerative diseases should not be considered as isolated rare entities only, but rather as a biologically interconnected group of disorders that converge on a limited number of fundamental pathogenic mechanisms. Second, despite their heterogeneity, a mechanistic and molecular classification provides a useful framework for understanding shared disease pathways, improving diagnosis, and identifying therapeutic opportunities. Third, early molecular characterization is increasingly central to clinical care, both for prognostic purposes and for access to targeted or emerging therapies.

Accordingly, the aim of this review is to provide a comprehensive and clinically relevant synthesis of current knowledge on neurodegenerative diseases in children. We focus on their epidemiology, molecular classification, key pathophysiological mechanisms, diagnostic approaches, and current as well as emerging therapeutic strategies. By bringing together these complementary perspectives, this review seeks to offer a structured overview of a complex and rapidly evolving field, while highlighting the importance of multidisciplinary and early precision-based approaches in pediatric neurodegeneration.

## 2. Materials and Methods

This article was designed as a narrative, comprehensive review of pediatric neurodegenerative diseases, with a particular focus on disorders characterized by progressive neurological decline, developmental regression, and genetically determined disruption of essential cellular pathways. The review was based on the assumption that pediatric neurodegenerative diseases, although clinically heterogeneous, can be meaningfully analyzed within an integrated framework combining epidemiology, molecular classification, pathophysiology, diagnosis, and therapeutic strategies.

A structured literature search was performed in PubMed/MEDLINE, Scopus, and Google Scholar to identify relevant publications addressing neurodegenerative diseases in children. Additional articles were identified through manual screening of reference lists from key reviews, consensus statements, and original research papers. The search strategy included combinations of the following keywords: pediatric neurodegeneration, childhood neurodegenerative diseases, lysosomal storage disorders, leukodystrophies, mitochondrial diseases, peroxisomal disorders, developmental regression, neuroinflammation, oxidative stress, gene therapy, precision medicine, diagnosis, and treatment. Emphasis was placed on articles published in English, with particular attention to studies published in the last 10 years, while older landmark articles were included when considered essential for historical context or mechanistic understanding.

The inclusion criteria comprised original research articles, systematic or narrative reviews, consensus statements, clinical guidelines, epidemiological studies, and translational studies relevant to pediatric neurodegenerative diseases. Publications were selected if they addressed at least one of the following domains: epidemiology, molecular or genetic classification, pathophysiological mechanisms, diagnostic approaches, or therapeutic interventions in children. Studies focusing predominantly on adult-onset neurodegenerative diseases were excluded unless they provided mechanistic insights directly relevant to pediatric disorders. Case reports with limited generalizability, non-peer-reviewed sources, and articles lacking sufficient methodological or clinical detail were also excluded, except where rare disorders required illustrative citation of limited pediatric data.

The final selection of references was based on clinical relevance, methodological quality, recency, and their contribution to the conceptual scope of the review. Priority was given to studies with clear diagnostic definitions, recognized disease classifications, robust clinical or molecular data, and direct applicability to pediatric populations. Because of the rarity and heterogeneity of these disorders, the evidence base includes both high-level reviews and selected disease-specific observational studies.

This review was not conducted as a formal systematic review or meta-analysis; therefore, no quantitative synthesis of effect sizes was performed. Instead, the objective was to provide a clinically oriented and mechanistically integrated overview of the field, highlighting convergent pathogenic pathways and emerging precision-based therapeutic strategies in pediatric neurodegeneration.

## 3. Epidemiology of Neurodegenerative Diseases in Children

Childhood neurodegenerative diseases are considered rare conditions, but they represent a significant burden on families and public health services. They are often described under different broader clinical umbrellas, such as progressive intellectual and neurological impairment (PIND) or infantile dementia, because many disorders share a common trajectory of a developmental plateau followed by loss of previously acquired skills, progressive motor impairment, seizures, and early mortality. Population-level estimates remain difficult because these conditions are heterogeneous, frequently ultra-rare, and often clinically underdiagnosed. Although genomic diagnostics and emerging disease registries have improved case ascertainment for some monogenic pediatric neurodegenerative disorders, particularly selected lysosomal diseases and leukodystrophies, this progress is not uniform across the field. Many disorders remain diagnostically challenging because of marked phenotypic variability, incomplete penetrance, probable effects of modifier genes and epigenetic factors, and the still-limited size of subtype-specific cohorts, which constrain robust genotype–phenotype correlations and risk prediction [1,2,3,4].

Estimated prevalence varies widely depending on the specific disorder and the population studied, ranging from 1 in 10,000 to less than 1 in 1,000,000 live births. Many conditions are more common in populations with high rates of consanguinity due to autosomal recessive inheritance patterns. Improved survival of affected infants and increased recognition of milder phenotypes have contributed to an apparent increase in prevalence in recent decades [5].

A useful “whole-field” estimate comes from a large scoping review of monogenic childhood dementia conditions, which identified 170 genetic disorders and modeled the collective burden using published incidence and survival data. The authors estimated that currently untreatable childhood dementias have a collective incidence of 34.5 per 100,000 births (≈1 in 2900), while additional treatable inborn errors of metabolism could contribute substantially to the burden if not detected early—raising the modeled total (treatable + untreatable) to 84.3 per 100,000 births (≈1 in 1190) in settings without adequate early detection and treatment. These figures emphasize that “rare” does not necessarily mean “negligible” at a population level when many disorders are grouped together [6].

### 3.1. Major Etiological Groups and Key Epidemiological Patterns

*(a) Leukodystrophies and Genetic White Matter Disorders (GWMD)*

Leukodystrophies are major contributors to pediatric neurodegenerative pathology, often presenting with developmental regression, spasticity, ataxia, seizures, and progressive loss of function. A clinical report from the American Academy of Pediatrics summarizes that leukodystrophies are common among rare diseases, citing an incidence of at least 1 in 4700 live births [7,8].

More granular regional epidemiology comes from a longitudinal population-based cohort in Northern Finland, where the cumulative childhood incidence of childhood-onset GWMDs was 30 per 100,000 live births, spanning both classic leukodystrophies and a broader spectrum of genetic leukoencephalopathies. Notably, a substantial share involved newly described or recently recognized genetic etiologies, underscoring the impact of improved molecular diagnostics on measured incidence.

More granular regional epidemiology comes from a longitudinal population-based cohort in Northern Finland, where the cumulative childhood incidence of childhood-onset GWMDs was 30 per 100,000 live births, spanning both classic leukodystrophies and a broader spectrum of genetic leukoencephalopathies. Notably, a substantial share involved newly described or recently recognized genetic etiologies, underscoring the impact of improved molecular diagnostics on measured incidence [9].

However, ascertainment may be further refined by WGS and related population-genetic approaches, particularly in geographically distinct populations, where founder variants, linked loci, and unresolved inherited etiologies may remain underrecognized. In addition, some inflammatory or autoimmune white-matter disorders may phenocopy genetic leukoencephalopathies, and incomplete exclusion of such mimics may affect epidemiologic classification and incidence estimates.

*(b) Neuronal ceroid lipofuscinoses (NCLs* *)*

NCLs (including ceroid lipofuscinosis neuronal 1 (CLN1), CLN2, CLN3, etc.) are lysosomal neurodegenerative disorders and are among the more recognizable pediatric neurodegeneration syndromes because of the typical combination of epilepsy, neuroregression, and (in many forms) vision loss. This phenotype reflects lysosomal storage-material accumulation in neurons of the brain and retina, with retinal and visual-pathway involvement contributing to vision loss, and cortical dysfunction with progressive neuronal injury contributing to epilepsy and regression. Oxidative stress and neuroinflammatory cascades further amplify neuronal damage. A clinical review in Journal of Pediatric Genetics notes that reported NCL incidence varies widely by geography and ancestry, ranging from 1.3 to 7 per 100,000 live births [10].

Subtype-specific estimates are increasingly available. For example, a nationwide study using the French hospital discharge database estimated the incidence of CLN2 disease at 0.99 per 100,000 live births (2017–2023) and also documented substantial diagnostic delay—an epidemiologic feature that can distort prevalence estimates and delay access to disease-modifying therapy [11].

*(c) Primary mitochondrial disorders*

Mitochondrial disease is a heterogeneous group of disorders that often manifests in childhood with multisystem involvement (neurologic regression, epilepsy, encephalopathy, movement disorders, cardiomyopathy). A classic epidemiologic review highlights that population-based studies over time have demonstrated mitochondrial disorders are more common than previously appreciated, and it synthesizes prevalence/incidence estimates across age groups and study designs [12].

In the genomic era, cohort studies and diagnostic-approach papers also report population incidence figures used in clinical epidemiology discussions—for instance, a Journal of Clinical Medicine cohort study notes an incidence on the order of ~1.6 per 5000 (as cited in their background), while emphasizing that case identification depends strongly on testing pathways and criteria [13].

### 3.2. Why Is Epidemiology Difficult in Pediatric Neurodegeneration?

Diagnostic mimics and phenocopies: some acquired inflammatory or autoimmune white-matter disorders may resemble genetic leukoencephalopathies on clinical and MRI grounds, and incomplete exclusion of these mimics may distort epidemiologic classification and incidence estimates.

Several factors complicate accurate epidemiologic measurements, including diagnostic delay and misclassification. Early symptoms may mimic more common developmental disorders or cerebral palsy-like presentations, while seizure-related manifestations must be interpreted within an age-dependent diagnostic framework. In the neonatal period, suspected seizures are classified according to a distinct EEG-centered framework and often require etiologic reassessment, whereas later in infancy and childhood some neurodegenerative disorders may resemble epilepsy syndromes or developmental epileptic encephalopathies [11,14].

- Changing case definitions: “Childhood dementia,” PIND, and disease-specific criteria are not always applied consistently across countries or over time [6].
- Founder effects and inbreeding are other elements because: incidence can be substantially higher in specific populations due to carrier frequency, founder variants, or consanguinity patterns [6].
- Changes in the level of confirmation with genomics and neonatal screening have allowed expanded access to next-generation sequencing and selective neonatal screening for certain conditions that can increase observed incidence (through detection), while changing survival and prevalence over time (through treatment) [7].

Overall, pediatric neurodegenerative diseases represent a “rare-but-many” epidemiologic landscape: each disorder may be uncommon, yet together they produce a measurable incidence and significant family and health-system impact. Continued improvements in surveillance, standardized definitions, equitable genomic diagnostics, and international registries are essential to refine incidence/prevalence estimates and to evaluate the population impact of emerging therapies.

## 4. Classification of Neurodegenerative Diseases in Children

Pediatric neurodegenerative diseases comprise a broad and heterogeneous group of conditions that cannot be fully captured by a single classificatory system. Depending on the perspective adopted, these disorders may be organized according to the primary molecular defect, the affected organelle or biochemical pathway, the predominant clinic-radiologic phenotype, the mode of inheritance, or the age at onset. Although these approaches are all useful, they are not conceptually equivalent and should not be merged indiscriminately within one linear taxonomy. Failure to distinguish between them may generate internal inconsistency and obscure clinically relevant relationships among disorders.

For this reason, the present review adopts a primary etiologic–molecular framework, in which pediatric neurodegenerative diseases are grouped according to the principal defective cellular system or biological pathway. Within this structure, major categories include lysosomal disorders, mitochondrial disorders, peroxisomal disorders, myelin-related and glial disorders, disorders of metal homeostasis, and other monogenic conditions affecting proteostasis, deoxyribonucleic acid (DNA) repair, cytoskeletal integrity, axonal transport, or synaptic function. This approach is particularly useful because it reflects underlying disease biology, facilitates mechanistic comparison across conditions, and aligns more directly with emerging precision-based therapeutic strategies.

At the same time, certain highly relevant clinical entities are better understood as clinic-radiologic or syndromic groupings rather than as uniform mechanistic classes. Leukodystrophies are a particularly important example. Although they are often discussed as a single disease family in pediatric neurology, they do not represent one coherent molecular category. Instead, they encompass inherited white-matter disorders arising from defects in multiple distinct biological systems, including lysosomal metabolism, peroxisomal lipid handling, mitochondrial function, astrocytic support, oligodendroglial homeostasis, and microglial signaling. Accordingly, leukodystrophies are best regarded as a clinically valuable phenotypic subgroup that overlaps with several etiologic classes rather than as a top-level mechanistic category equivalent to lysosomal, mitochondrial, or peroxisomal disease [15,16,17,18,19,20,21,22,23,24,25,26,27,28,29,30,31,32,33,34,35,36].

A similar caution applies to other commonly used labels, such as autophagy–lysosome dysfunction, DNA repair disorders, repeat expansion disorders, and ion channel or synaptic disorders. In addition, repeat expansion disorders should not be viewed only as a mutational architecture grouping, because their downstream pathogenic effects may include toxic RNA or protein gain-of-function, abnormal protein misfolding, and aggregation, thereby linking them to broader convergent mechanisms of neurodegeneration.

Although these labels capture important pathogenic mechanisms, mutational architectures, or neurophysiologic consequences, they do not operate at the same conceptual level. Some refer to cross-cutting mechanistic pathways, whereas others reflect genetic architecture groupings rather than primary disease classes. Pediatric neurodegenerative diseases are therefore best understood within a multidimensional classification framework. In this review, the primary organizational principle is etiologic–molecular, complemented by clinico-radiologic syndromes, cross-cutting mechanistic pathways, and genetic architecture groupings, as summarized in Table 1 [37,38,39,40,41,42,43,44,45,46,47,48,49,50].

Within this framework, the principal etiologic–molecular groups relevant to pediatric neurodegeneration include disorders of the lysosome, mitochondrion, peroxisome, and myelin-related or glial systems, among others. By contrast, some commonly invoked categories are better regarded as secondary or intersecting frameworks rather than directly parallel top-level classes.

For example, although leukodystrophies are often discussed as a discrete disease family in pediatric neurology, they are more appropriately regarded as a clinico-radiologic syndrome rather than a single etiologic–molecular category. Inherited white-matter degeneration may result from defects in multiple distinct biological systems, including lysosomal, peroxisomal, mitochondrial, astrocytic, oligodendroglial, and microglial pathways. Leukodystrophies are therefore best understood as a cross-category clinico-radiologic grouping rather than as a mechanistically uniform disease class.

### 4.1. Lysosomal Disorders

Lysosomal disorders represent a major class of pediatric neurodegenerative diseases and are caused by defects in lysosomal enzymes, membrane proteins, activator proteins, or intracellular trafficking pathways. These abnormalities lead to impaired degradation of macromolecules and progressive intralysosomal substrate accumulation. Neurons are especially vulnerable because they depend heavily on the continuous turnover of their membrane components, organelles, and protein aggregates. In many lysosomal disorders, neurodegeneration reflects not only toxic storage but also broader dysfunction of the autophagy–lysosome system, with secondary impairment of proteostasis, mitochondrial homeostasis, axonal transport, and neuroinflammatory signaling. Representative disorders include Tay–Sachs disease, Sandhoff disease, metachromatic leukodystrophy, Krabbe disease, Niemann–Pick disease type C, and the neuronal ceroid lipofuscinoses. Clinically, these conditions commonly manifest with developmental regression, epilepsy, movement abnormalities, visual loss, spasticity, and progressive cognitive decline, often in association with systemic features such as organomegaly or skeletal involvement [15,16,22,29,30,31,32,33,34,35,36].

### 4.2. Primary Mitochondrial Disease: Genetic and Diagnostic Framework

Primary mitochondrial diseases comprise a heterogeneous group of inherited disorders caused by pathogenic variants in mitochondrial DNA or in nuclear genes required for mitochondrial structure, replication, translation, respiratory-chain assembly, cofactor metabolism, and organelle maintenance. Rather than representing a single clinical entity, these disorders form a broad genetic and biochemical spectrum unified by impaired oxidative phosphorylation and deficient cellular energy production. Additional mechanisms, including altered redox signaling, abnormal calcium homeostasis, defective mitochondrial protein synthesis, and impaired organelle quality control, may further contribute to tissue injury.

In childhood, primary mitochondrial disease is particularly relevant because the developing nervous system depends heavily on continuous ATP production. Consequently, affected patients may present with developmental delay, developmental regression, epilepsy, encephalopathy, movement disorders, ataxia, hypotonia, ophthalmologic abnormalities, or multisystem disease involving the heart, liver, skeletal muscle, endocrine system, or gastrointestinal tract. However, these manifestations are diagnostically nonspecific. Several other inherited metabolic diseases, including organic acidemias, fatty acid oxidation disorders, peroxisomal disorders, lysosomal diseases, and congenital disorders of glycosylation, can produce overlapping neurologic and systemic phenotypes.

For this reason, primary mitochondrial disease should not be diagnosed solely on the basis of clinical similarity. A robust diagnostic approach requires integration of clinical history, neurologic examination, metabolic biomarkers, neuroimaging, enzymatic respiratory-chain studies when indicated, and molecular genetic testing. Particular attention should be given to inheritance pattern, tissue specificity, age at onset, episodic decompensation, and evidence of multisystem involvement [12,13,50,51,52,53,54,55].

### 4.3. Peroxisomal Disorders

Peroxisomal disorders arise either from defects in peroxisome biogenesis or from deficiencies of individual peroxisomal enzymes or transporters. These abnormalities impair very-long-chain fatty acid metabolism, branched-chain fatty acid processing, plasmalogen synthesis, and oxidative homeostasis. In the nervous system, disturbed lipid metabolism affects membrane composition, myelination, and axonal integrity, while secondary interactions with mitochondrial pathways may further amplify cellular dysfunction. Zellweger spectrum disorders, X-linked adrenoleukodystrophy, and D-bifunctional protein deficiency are representative examples. Neurologic manifestations commonly include hypotonia, seizures, leukodystrophy, developmental delay, visual and hearing impairment, and, in selected disorders, adrenal insufficiency. From a classificatory perspective, these diseases form a coherent etiologic group even though some of them also fall within the clinic-radiologic spectrum of leukodystrophies [56,57,58,59,60,61,62,63,64,65].

### 4.4. Myelin-Related and Glial Disorders

A further major category includes disorders in which neurodegeneration is driven primarily by defective myelin formation, impaired myelin maintenance, or disturbed glial support of white-matter integrity. This group includes primary hypomyelinating and demyelinating disorders as well as astrocytopathies and microgliopathies in which white-matter degeneration emerges through non-neuronal mechanisms. Examples include Canavan disease, Pelizaeus–Merzbacher disease, Alexander disease, vanishing white matter disease, and selected microglial leukodystrophies. The common biological theme is that progressive neurologic decline may result not only from intrinsic neuronal defects but also from failure of oligodendrocyte, astrocyte, or microglial homeostasis. Clinically, affected children often develop progressive motor decline, spasticity, ataxia, seizures, bulbar symptoms, or cognitive deterioration, with brain MRI playing a central role in pattern recognition and subclassification [7,37,38,39,40,41,42,43,44,45,46,47,48,49,66].

### 4.5. Disorders of Metal Homeostasis and Selective Basal Ganglia Vulnerability

Certain pediatric neurodegenerative diseases are best grouped according to highly characteristic metabolic disturbances affecting trace metal homeostasis, especially iron handling. Neurodegeneration with brain iron accumulation represents the prototypical example, encompassing conditions such as pantothenate kinase-associated neurodegeneration and PLA2G6-associated neurodegeneration. Although genetically heterogeneous, these disorders share abnormal iron deposition in the basal ganglia, progressive extrapyramidal dysfunction, dystonia, parkinsonism, spasticity, and cognitive decline. Their inclusion as a distinct group is justified by the combination of a recognizable biochemical theme and a relatively specific neuroanatomical pattern of vulnerability [67,68,69,70,71,72,73,74,75,76].

### 4.6. Other Monogenic Neurodegenerative Disorders

Beyond the major organelle-based and myelin-related groups, a substantial number of pediatric neurodegenerative diseases arise from other monogenic mechanisms, including impaired proteostasis, defective DNA repair and genome maintenance, cytoskeletal instability, axonal transport failure, and synaptic dysfunction. Examples include ataxia-telangiectasia, Cockayne syndrome, Aicardi–Goutières syndrome, selected hereditary spastic paraplegias, juvenile Huntington disease, dentatorubral-pallidoluysian atrophy, and some developmental and epileptic encephalopathies with progressive regression. These conditions do not always fit cleanly into a single organelle-centered framework, but they are mechanistically important because they highlight additional routes by which inherited defects can lead to progressive neuronal dysfunction and neurodegeneration in childhood [77,78,79].

### 4.7. Cross-Cutting Clinico-Radiologic and Mechanistic Groupings

Although the etiologic–molecular framework provides the structural basis for this review, several cross-cutting groupings remain clinically indispensable. These include leukodystrophies, epileptic neurodegenerative encephalopathies, developmental regression syndromes, movement disorder–predominant neurodegenerative conditions, and disorders with combined neurodevelopmental and neurodegenerative phenotypes. Such categories are especially useful in clinical practice because they reflect the way patients present diagnostically rather than the way diseases are ultimately classified molecularly. Likewise, pathogenic themes such as lysosomal-autophagic failure, mitochondrial quality-control disruption, neuroinflammation, oxidative stress, ferroptotic vulnerability, or axonal transport impairment cut across multiple etiologic groups and help explain both phenotypic overlap and shared therapeutic challenges.

Taken together, these considerations indicate that classification in pediatric neurodegeneration should be understood as hierarchical and multidimensional, not flat or uniform. A biologically informed primary classification based on the principal defective cellular pathway offers conceptual clarity and translational relevance, while clinic-radiologic and mechanistic groupings remain essential complementary tools for diagnosis and comparative interpretation. This integrated approach is particularly appropriate in a field where similar phenotypes may emerge from distinct molecular defects, and conversely, where perturbation of the same pathway may generate diverse clinical presentations depending on developmental timing, tissue vulnerability, and residual function [7,37,42,70,71,72,73,74,75,76,79,80,81,82,83,84,85,86,87,88,89,90,91,92,93,94,95,96].

In particular, autophagy-related dysfunction should be interpreted as a shared pathogenic mechanism that cuts across multiple pediatric neurodegenerative disorders rather than as a mechanistically independent top-level disease category. This distinction is important when moving from conceptual classification to disease-specific discussion. Behavioral and psychiatric manifestations should likewise be viewed as clinically relevant cross-cutting features rather than disease-defining top-level classes, since they may appear early in several pediatric neurodegenerative disorders but are generally insufficiently specific for etiologic classification.

Progressive neurodegeneration is often accompanied by organomegaly, skeletal abnormalities, and visual impairment [15,16].

LSDs are usually caused by inherited pathogenic variants. Most follow an autosomal recessive pattern of inheritance, in which each parent typically carries one pathogenic allele and the affected child inherits two pathogenic alleles. A smaller number are X-linked and therefore tend to affect hemizygous males more severely.

Table 2 provides a comparative overview of selected pediatric lysosomal storage disorders (LSDs), integrating enzymatic deficiency, genetic background, inheritance pattern, and principal clinical manifestations within a unified framework. By aligning the defective lysosomal enzyme with its corresponding causative gene (e.g., *GBA1*, *hexosaminidase subunit alpha (HEXA)*, *SMPD1/NPC1/NPC2*, *GAA*, *IDUA*), the table highlights the direct relationship between molecular dysfunction and intracellular substrate accumulation. This genotype–biochemical defect axis underlies the progressive cellular pathology characteristic of LSDs.

All listed disorders follow an autosomal recessive inheritance pattern, emphasizing the importance of carrier detection, genetic counseling, and family-based molecular screening strategies. The table also illustrates how differences in substrate type—glycosphingolipids, gangliosides, sphingomyelin, glycogen, or glycosaminoglycans—translate into tissue-specific vulnerability.

For example, neuronal lipid accumulation predominantly results in progressive neurodegeneration (e.g., Tay–Sachs disease) through disruption of membrane biophysics, intracellular trafficking, and cell signaling, with secondary oxidative stress and neuroinflammation further contributing to neuronal injury. Accumulated lipids may additionally disrupt membrane fluidity, alter lipid raft–dependent signaling, and impair calcium homeostasis. In selected disorders, such as Krabbe disease, psychosine exerts direct cytotoxic effects that further aggravate neuronal and oligodendroglial injury. By contrast, glycogen storage in muscle cells leads to cardiomyopathy and respiratory compromise (e.g., Pompe disease), whereas macrophage lipid engorgement in Gaucher disease contributes to hepatosplenomegaly and bone pathology.

By juxtaposing molecular defects with systemic manifestations, Table 2 summarizes genotype–phenotype associations and highlights disease heterogeneity, both of which are relevant to clinical interpretation, prognosis assessment, and therapeutic decision-making. This integrative perspective is particularly relevant in the era of targeted therapies—including enzyme replacement therapy, substrate reduction therapy (SRT), and emerging gene-based approaches—where precise molecular diagnosis directly informs clinical management.

### 4.8. Major Lysosomal Storage Disorders in Children

Major lysosomal storage disorders frequently manifest in childhood and are associated with progressive multisystem involvement, including hematologic, skeletal, muscular, and central nervous system disease.

#### 4.8.1. Gaucher Disease

Gaucher disease is caused by pathogenic variants in the *GBA1* gene, resulting in glucocerebrosidase deficiency and subsequent accumulation of glucocerebroside in macrophages, forming characteristic Gaucher cells. Although genotype–phenotype relationships are not absolute, illustrative associations have been reported; for example, at least one N370S allele is usually associated with non-neuronopathic type 1 Gaucher disease, whereas L444P, especially in homozygosity, is more frequently linked to neuronopathic forms. Clinical manifestations include hepatosplenomegaly, cytopenias, bone crises, and fatigue. Disease severity varies widely depending on genotype and subtype [17,18].

#### 4.8.2. Tay–Sachs Disease

Tay–Sachs disease results from pathogenic variants in the *HEXA* gene encoding the α-subunit of hexosaminidase A. The enzymatic defect leads to GM2 ganglioside accumulation within neurons, causing progressive neurodegeneration. The infantile form is characterized by developmental regression, hypotonia, seizures, and early mortality. Recent advances focus on enzyme replacement and gene therapy approaches [19,20,21].

#### 4.8.3. Niemann–Pick

Niemann–Pick disease types A and B arise from pathogenic variants in SMPD1 leading to acid sphingomyelinase deficiency, whereas type C is caused by pathogenic variants in NPC1 or NPC2, impairing intracellular cholesterol trafficking. These defects result in lysosomal lipid accumulation and progressive neurovisceral involvement. In Niemann–Pick disease type C, neurological manifestations commonly include progressive cognitive decline, ataxia, and other features of worsening neurodegeneration, often in association with hepatosplenomegaly in pediatric patients [22,23].

#### 4.8.4. Pompe Disease

Pompe disease is caused by pathogenic variants in the *GAA* gene, resulting in acid α-glucosidase deficiency and lysosomal glycogen accumulation, particularly in muscle tissue. The infantile-onset form presents with hypotonia, hypertrophic cardiomyopathy, and respiratory insufficiency, whereas late-onset Pompe disease more typically manifests with progressive skeletal-muscle weakness and exercise intolerance, with myalgia reported in a proportion of patients. Early diagnosis and enzyme replacement therapy significantly improve outcomes [22,24,25].

#### 4.8.5. Hurler Syndrome (Mucopolysaccharidosis Type I)

Hurler syndrome (MPS I) is caused by pathogenic variants in the *IDUA* gene leading to α-L-iduronidase deficiency and accumulation of dermatan sulfate and heparan sulfate. Clinical features include coarse facial features, dysostosis multiplex, organomegaly, and neurocognitive decline. Advances in hematopoietic stem cell transplantation (HCST) and enzyme replacement therapy have improved survival and quality of life [26,27,28].

Advances in molecular diagnostics, particularly next-generation sequencing and expanded newborn screening programs, have enabled earlier and more precise identification of pathogenic variants, facilitating timely intervention. Therapeutic strategies have evolved substantially over the past two decades; enzyme replacement therapy (ERT) provides exogenous recombinant enzymes to restore lysosomal function in select disorders, while SRT, pharmacological chaperones, and emerging gene therapy approaches aim to address the underlying metabolic imbalance at a molecular level. Collectively, these innovations have transformed the clinical trajectory of several LSDs from rapidly progressive and life-limiting conditions to chronic, increasingly manageable diseases, significantly improving survival, functional outcomes, and health-related quality of life in pediatric populations [29,30,31,32,33,34,35,36].

### 4.9. Leukodystrophies

Leukodystrophies in children (Table 3) are a clinically and genetically diverse group of inherited white-matter disorders in which the primary pathology involves abnormal myelin formation (hypomyelination), defective myelin maintenance, or progressive myelin loss (demyelination), leading to impaired saltatory conduction and widespread network dysfunction.

Mechanistically, pediatric leukodystrophies can arise from defects intrinsic to oligodendrocytes (the myelinating cells), from astrocyte- and microglia-driven “glial leukodystrophies” that secondarily disrupt myelin homeostasis, or from metabolic derangements (e.g., lysosomal or peroxisomal dysfunction) that create a toxic biochemical milieu for white-matter integrity [7,37].

In Krabbe disease (globoid cell leukodystrophy), deficiency of galactocerebrosidase (*GALC*) results in psychosine accumulation, severe neuroinflammation, and oligodendroglial loss. In metachromatic leukodystrophy (MLD), deficiency of arylsulfatase A (ARSA) leads to sulfatide accumulation in myelin-forming and immune cells, causing progressive central and peripheral demyelination and neurodegeneration [38,39].

In vanishing white matter (VWM) disease, biallelic *EIF2B1–EIF2B5* variants disrupt translational control during cellular stress, producing a characteristic stress-vulnerable white-matter phenotype and progressive neurologic decline.

Clinically, children often present with developmental delay or regression, spasticity/ataxia, seizures, visual impairment, peripheral neuropathy, and multisystem involvement depending on the underlying pathway; brain MRI pattern recognition remains pivotal for phenotypic stratification and targeted molecular confirmation. Importantly, the therapeutic landscape is shifting from supportive care toward disease modification: genotype-informed early diagnosis enables time-sensitive interventions such as HCST in selected leukodystrophies and expanding gene-therapy strategies [including adeno-associated virus (AAV) -based approaches] aimed at restoring missing functions or correcting downstream inflammatory and metabolic cascades [40,41,42].

Table 3 summarizes selected inherited white matter disorders, highlighting inheritance patterns, causative genes (italicized according to HGNC nomenclature), principal pathobiological mechanisms, characteristic pediatric manifestations, and current or emerging disease-modifying therapeutic approaches. These disorders represent a genetically heterogeneous group that primarily affects central nervous system white matter through defects in myelin formation, maintenance, or glial homeostasis. The underlying pathogenic mechanisms include lysosomal dysfunction (e.g., *GALC*, *ARSA*), peroxisomal impairment (*ABCD1*), dysregulation of cellular stress responses (*EIF2B1–EIF2B5*), microglial signaling abnormalities (*CSF1R*), and astrocytic cytoskeletal aggregation (*GFAP*). Therapeutic strategies such as HSCT and gene therapy appear to provide the greatest benefit when implemented at presymptomatic or early symptomatic stages [43,44,45,46,47,48,49].

### 4.10. Mitochondrial Disorders

Mitochondria are essential organelles responsible for cellular energy production, regulation of apoptosis, calcium balance, and reactive oxygen species (ROS) homeostasis. Defects in mitochondrial function, caused by pathogenic variants in mitochondrial DNA (mtDNA) or nuclear genes encoding mitochondrial proteins, can profoundly impair neuronal metabolism and survival, especially in children whose nervous systems are still developing. Mitochondrial disorders in pediatric populations often manifest with neurodegenerative features due to the high energy demands of the brain and the vulnerability of neurons to oxidative stress and bioenergetic failure [13,51].

Examples include Leigh syndrome, MELAS (mitochondrial encephalomyopathy, lactic acidosis, and stroke-like episodes), and primary mitochondrial cytopathies, which present progressive neurologic decline, ataxia, epilepsy, and cognitive regression. Although extensive research has elucidated mitochondrial dysfunction in adult diseases such as Alzheimer’s and Parkinson’s, increasing evidence suggests that disrupted mitochondrial dynamics, mitophagy, and electron transport chain (ETC) activity contribute importantly to pediatric neurodegenerative phenotypes. However, the causal direction is not uniform across disorders: in primary mitochondrial diseases these abnormalities may represent upstream pathogenic drivers, whereas in other neurodegenerative conditions they may arise secondarily from protein or substrate accumulation, impaired proteostasis, lysosomal dysfunction, calcium dysregulation, and oxidative stress [52,53].

Primary mitochondrial disorders disrupt energy production by compromising the ETC and oxidative phosphorylation, leading to reduced adenosine triphosphate (ATP) generation and increased ROS. In the developing brain, this energy deficit accelerates neuronal dysfunction and cell death. Moreover, abnormalities in mitochondrial dynamics (fusion/fission balance) and defective mitophagy contribute to the accumulation of damaged mitochondria, further exacerbating neurodegenerative processes [54].

Advances in molecular genetics and metabolic profiling have improved the diagnosis and understanding of these disorders, but effective disease-modifying therapies remain limited. Current research emphasizes enhancing mitochondrial resilience, optimizing quality-control pathways such as mitophagy, and identifying early biomarkers to predict disease progression—critical steps toward tailored interventions in pediatric neurodegeneration [55].

More broadly, mitochondrial dysfunction has also been implicated in selected neurodevelopmental conditions, including ADHD and autism spectrum disorder, although these entities are not primarily classified as pediatric neurodegenerative diseases. This overlap further supports the view that impaired bioenergetics and mitochondrial quality control may influence a wider spectrum of childhood neurologic phenotypes.

In mtDNA-related disease, heteroplasmy and the threshold effect are central determinants of tissue specificity and phenotypic variability, helping explain why the same mtDNA variant may produce different manifestations across patients and tissues.

Table 4 summarizes representative pediatric neurodegenerative diseases in which mitochondrial dysfunction plays a central pathogenic role. Although these conditions differ in genetic origin —ranging from mtDNA mutations to nDNA defects—they share a common mechanism: impaired oxidative phosphorylation and defective ATP production. Because the developing brain has exceptionally high metabolic demands, even partial deficiencies in ETC activity or mitochondrial protein synthesis can lead to rapid neuronal dysfunction and progressive neurodegeneration.

It is important to note that phenotypic overlaps are common among mitochondrial disorders. Clinical manifestations such as psychomotor regression, epilepsy, ataxia, and lactic acidosis are not disease-specific but reflect systemic bioenergetic failure. Additionally, genotype–phenotype correlations may vary considerably due to heteroplasmy, tissue-specific energy requirements, and environmental modifiers.

Therefore, the disorders listed in the table should be understood as examples within a broader spectrum of pediatric mitochondrial encephalopathies, all unified by primary or secondary mitochondrial dysfunction as a driving mechanism of neurodegeneration. Specific pediatric mitochondrial disorders may overlap in clinical presentation but share mitochondrial energy failure as a core mechanism.

### 4.11. Peroxisomal Disorders: Molecular Mechanisms and Clinical Perspectives

Peroxisomes are dynamic, single-membrane-bound organelles that play essential roles in cellular lipid metabolism, redox homeostasis, and biosynthetic pathways. They are responsible for β-oxidation of very long-chain fatty acids (VLCFAs), α-oxidation of branched-chain fatty acids, plasmalogen biosynthesis, bile acid synthesis, and ROS detoxification. Disruption of peroxisomal biogenesis or single peroxisomal enzyme functions results in a heterogeneous group of inherited metabolic diseases collectively referred to as peroxisomal disorders [56,57].

Peroxisomal disorders are broadly classified into (a) peroxisome biogenesis disorders (PBDs) and (b) single peroxisomal enzyme or transporter deficiencies. PBDs arise from pathogenic variants in *PEX* genes encoding peroxins—proteins required for peroxisome assembly, matrix protein import, and membrane formation. The PBD spectrum includes disorders within the Zellweger spectrum, with Zellweger syndrome representing the most severe phenotype. These conditions are characterized by multisystem involvement, including profound neurological impairment, hepatic dysfunction, and developmental abnormalities. At the cellular level, impaired peroxisomal matrix protein imports lead to “empty” peroxisomal membrane structures and widespread metabolic dysregulation [58,59].

In contrast, single enzyme deficiencies typically affect specific metabolic pathways. A paradigmatic example is X-linked adrenoleukodystrophy, caused by pathogenic variants in the *ABCD1* gene encoding a peroxisomal membrane transporter responsible for VLCFA import. Accumulation of saturated VLCFAs contributes to inflammatory demyelination in the central nervous system and adrenal insufficiency. Another example is Refsum disease, which results from defective α-oxidation of phytanic acid due to pathogenic variants in *PHYH* or *PEX7*, leading to progressive neuropathy and retinopathy [60,61].

Mechanistically, the pathophysiology of peroxisomal disorders extends beyond substrate accumulation. Perturbed lipid homeostasis affects membrane composition, myelination, and mitochondrial function. Increasing evidence highlights crosstalk between peroxisomes and mitochondria in fatty acid oxidation and ROS regulation, suggesting that secondary mitochondrial dysfunction contributes to disease progression. Furthermore, altered plasmalogen levels impair neuronal membrane integrity and synaptic function, providing a mechanistic link to the severe neurodevelopmental phenotypes observed in PBDs [62,63].

Recent advances in next-generation sequencing have significantly improved diagnostic accuracy, enabling early detection and genotype–phenotype correlation analyses. Newborn screening for X-linked adrenoleukodystrophy using dried blood spot VLCFA measurements has facilitated presymptomatic identification and early intervention [64,65].

Therapeutic approaches remain limited and are largely supportive; however, HCST and gene therapy have shown promise in selected phenotypes of X-linked adrenoleukodystrophy. Ongoing research into small-molecule chaperones, metabolic bypass strategies, and targeted gene editing holds potential for broader therapeutic applicability [66]. A concise overview of major peroxisomal disorders is provided in Table 5.

In summary, peroxisomal disorders represent a complex group of inherited metabolic diseases characterized by impaired lipid metabolism and systemic involvement. Continued elucidation of peroxisomal biology, inter-organelle communication, and lipid signaling pathways will be essential for identifying novel biomarkers and developing targeted therapeutic strategies.

### 4.12. Other Genetic Neurodegenerative Conditions

This category includes disorders related to impaired protein degradation, cytoskeletal dysfunction, or synaptic abnormalities, such as spinal muscular atrophy, neuronal ceroid lipofuscinoses, and certain forms of hereditary spastic paraplegia [67,68,69].

## 5. Pathophysiological Mechanisms in Neurodegenerative Diseases in Children

Childhood neurodegenerative diseases are predominantly genetic and often intersect with ongoing brain development (synaptogenesis, myelination, circuit refinement). As a result, “degeneration” frequently reflects a combination of toxic substrate accumulation, failure of organelle quality control, selective neuronal/glial vulnerability, and secondary cascades (inflammation, oxidative injury, excitotoxicity) that amplify primary molecular defects [70,71].

In pediatric neurodegenerative diseases, neurodevelopment and neurodegeneration should not be viewed as entirely separate processes. In many monogenic conditions, the primary molecular defect is likely to affect the nervous system from early developmental stages, altering neuronal differentiation, synaptic maturation, axonal growth, circuit assembly, or glial support before overt regression becomes clinically apparent. These early abnormalities may not immediately cause neuronal loss, but they can generate a structurally and functionally vulnerable neural substrate with reduced capacity to tolerate subsequent metabolic stress, toxic substrate accumulation, mitochondrial dysfunction, or neuroinflammation. In this framework, later neurodegeneration may represent the progressive consequence of both defective maintenance and abnormal developmental wiring, rather than a purely post-developmental process. This developmental–degenerative continuum is particularly relevant in childhood disorders, where the brain is still undergoing active synaptogenesis, myelination, and network refinement at the time disease mechanisms begin to act. Recognizing this interaction adds important mechanistic depth to pediatric neurodegeneration and helps explain why the same genetic defect may produce mixed phenotypes involving developmental delay, regression, and progressive neurological decline [72].

**Figure 1:**
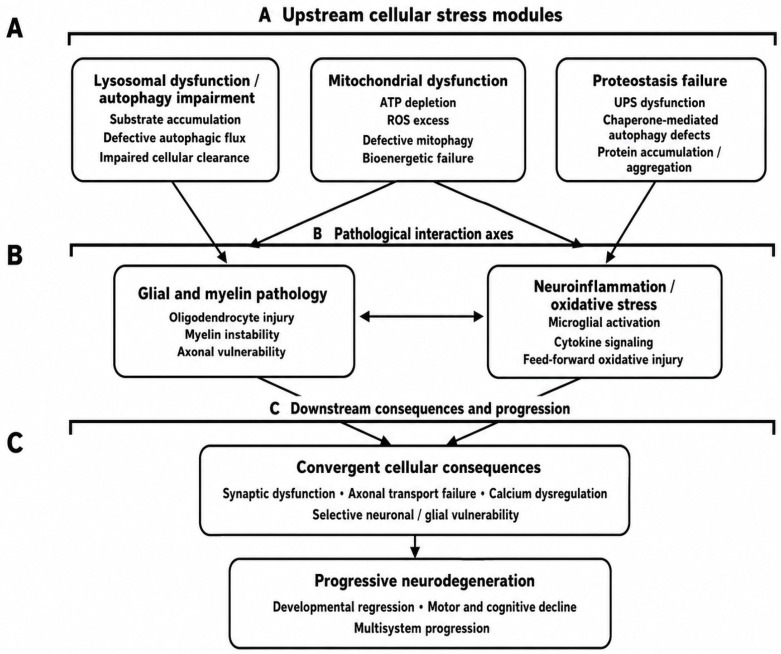
Schematic Overview of Convergent Cellular Mechanisms Involved in Pediatric Neurodegeneration. **Legend:** A = Upstream cellular stress modules; B = Pathological interaction axes; C = Downstream consequences and progression; ATP, adenosine triphosphate; ROS, reactive oxygen species; UPS, ubiquitin–proteasome system.

The major shared molecular pathways underlying pediatric neurodegenerative disorders are summarized schematically in Figure 1.

Schematic overview of convergent cellular mechanisms involved in pediatric neurodegeneration. Lysosomal dysfunction/autophagy impairment, mitochondrial dysfunction, and proteostasis failure represent major upstream pathogenic processes that promote glial and myelin pathology and neuroinflammation/oxidative stress. Their interplay results in shared downstream cellular consequences, including synaptic dysfunction, axonal transport failure, calcium dysregulation, and selective neuronal/glial vulnerability, which together drive progressive neurodegeneration characterized by developmental regression, motor and cognitive decline, and multisystem progression.

### 5.1. Insufficiency of the Lysosomal-Autophagic System (Impaired Elimination and Toxic Storage)

A large proportion of pediatric neurodegenerative diseases arise from LSDs, in which deficient lysosomal enzymes/transporters or trafficking factors cause intralysosomal substrate accumulation and dysfunction of the broader autophagy-lysosome pathway.

Mechanistically, lysosomal failure produces:

- Blocked autophagic flux (inefficient delivery/degradation of damaged proteins/organelles), promoting proteotoxic stress and organelle dysfunction [70].
- Disrupted lysosomal biogenesis and cellular stress responses governed by transcription factor EB (TFEB) and related transcriptional programs contribute to impaired autophagy–lysosome function; under physiological conditions, these pathways coordinate nutrient sensing with lysosomal capacity and autophagic flux [73].
- Secondary synaptic and axonal pathology, as neurons rely heavily on long-range transport and continuous turnover of vesicles, mitochondria, and membrane proteins; impaired clearance preferentially injures distal axons/synapses first [74].

In addition to lysosomal dysfunction, broader impairment of proteostasis is increasingly recognized in pediatric neurodegeneration. The ubiquitin–proteasome system, macroautophagy, and chaperone-mediated autophagy cooperate to maintain neuronal protein quality control, and disruption of these pathways may lead to abnormal protein accumulation, aggregation, and progressive cellular injury. This framework is relevant across multiple pediatric disease categories, including lysosomal disorders, neuronal ceroid lipofuscinoses, and leukodystrophies, and helps unify mechanistic links between defective clearance and neurodegeneration [75].

An additional emerging concept is that lysosomal substrate accumulation may, in selected disorders, have biophysical consequences beyond simple bulk storage, potentially involving condensed or aggregate-like states that further impair lysosomal homeostasis and proteostasis [75,76]. Although this concept remains preliminary and is not established as a universal feature of lysosomal storage diseases, it offers a useful framework for understanding progressive cellular irreversibility.

### 5.2. Myelin/Oligodendrocyte Pathology and Lipid Dysmetabolism (Leukodystrophy Mechanisms)

In many pediatric leukodystrophies, the primary lesion is not neuronal protein aggregation but glial metabolic failure—especially in oligodendrocytes, which must synthesize vast amounts of membrane and myelin lipids. Shared cellular themes include defects in lipid handling, protein production/ER load, and organelle stress within oligodendrocytes, leading to hypomyelination/demyelination and downstream axonal degeneration [77].

A prototypical example is Krabbe disease (globoid cell leukodystrophy), where GALC deficiency leads to accumulation of the cytotoxic sphingolipid psychosine, which perturbs membrane architecture, signaling, and cell viability—driving oligodendrocyte loss, demyelination, and a strong inflammatory response that accelerates neurodegeneration [38,48,71,78].

### 5.3. Mitochondrial Bioenergetic Failure and Impaired Mitochondrial Quality Control

Many severe early-onset neurodegenerative phenotypes (e.g., Leigh spectrum) reflect oxidative phosphorylation impairment, producing chronic ATP shortage in energy-demanding neural tissue and altering redox balance, calcium buffering, and apoptotic thresholds [79].

Key mitochondrial-driven mechanisms include:

- Energy failure at synapses and nodes → impaired neurotransmission, axonal maintenance, and ionic homeostasis.
- ROS overproduction and redox signaling imbalance → oxidative damage to lipids, proteins, and mtDNA, feeding forward into further respiratory chain dysfunction [80,81].
- Defective mitochondrial quality control, including impaired mitophagy and disturbed biogenesis coupling, may increase the burden of dysfunctional mitochondria—particularly damaging in long-lived, post-mitotic neurons [80,82].
- Accumulation of dysfunctional mitochondria may then lead to reduced ATP production and energy failure at synapses and nodes, impairing neurotransmission, axonal maintenance, and ionic homeostasis.
- The same process may also promote ROS overproduction and redox imbalance, causing oxidative damage to lipids, proteins, and mtDNA and further amplifying respiratory-chain dysfunction [51,81,83].
- In mtDNA-related disorders, the severity and tissue distribution of bioenergetic failure are further shaped by heteroplasmy, threshold effects, and random segregation of mtDNA variants across cells and tissues. These principles help explain why the same mtDNA variant may produce marked variability in penetrance, organ selectivity, and clinical phenotype.

### 5.4. Neuroinflammation and Maladaptive Glial Responses

Microglia and astrocytes often shift from homeostatic roles (synapse pruning, debris clearance, trophic support) toward chronic activation that can become neurotoxic. In pediatric disorders, inflammation can be triggered directly by stored substrates (danger signals), dying oligodendrocytes/neurons, or altered lipid species; once established, it amplifies degeneration through cytokines, ROS/RNS, complement, and excitatory signaling changes [71,84].

Conceptually, neuroinflammation contributes via:

- Induction/sensing/transduction/effectors (pattern-recognition receptors, inflammasomes, NF-κB programs) that converge across neurodegenerative conditions [85].
- Non–cell-autonomous toxicity, where stressed glia worsens neuronal survival (e.g., inflammatory demyelination harming axons) [86,87].

### 5.5. Oxidative Stress as a Feed-Forward Amplifier

Oxidative stress is frequently downstream of mitochondrial impairment and inflammation, but it can become a self-sustaining injury loop: ROS/RNS damage membranes (including myelin), inactivate enzymes, and destabilize cytoskeletal/transport proteins—worsening axonal transport and metabolic function [76,88,89].

### 5.6. Excitotoxicity, Calcium Dysregulation and Ferroptosis/Oxytosis

In multiple pediatric neurodegenerative contexts, energy failure and oxidative injury reduce the capacity to maintain ionic gradients and glutamate handling. This promotes excessive glutamatergic signaling, pathological calcium influx, mitochondrial overload, and activation of degradative enzymes [82,90,91].

In parallel, oxidative stress and iron/lipid peroxidation pathways can engage ferroptosis-like mechanisms, particularly relevant to lipid-rich white matter and developing brain regions with dynamic metabolic demands [82,92].

### 5.7. Axonal Transport Failure and “Dying-Back” Neurodegeneration

Because neurons depend on long-distance trafficking of mitochondria, vesicles, RNA/protein complexes, and autophagic cargos, disruptions in microtubule-based transport produce distal axon/synapse vulnerability. In pediatric diseases, transport can be impaired by [76,93,94]:

- Organelle dysfunction (energy deficits limiting motor activity),
- Oxidative modification of transport machinery,
- Inflammatory mediators altering cytoskeletal dynamics, accumulated undegraded cargos overwhelming trafficking routes.

### 5.8. Peroxisomal Dysfunction and Toxic Very-Long-Chain Lipid Accumulation

Peroxisomes are central to very-long-chain fatty acid (VLCFA) metabolism and myelin lipid homeostasis; peroxisomal defects can injure oligodendrocytes and destabilize myelin, secondarily harming axons. In childhood cerebral X-linked adrenoleukodystrophy, VLCFA-related membrane stress and downstream inflammatory demyelination represent a major pathophysiological axis [95,96].

### 5.9. Integrative Overview of Major Pathophysiological Mechanisms

Although pediatric neurodegenerative diseases are genetically and biochemically heterogeneous, several core molecular mechanisms recur across disorders and converge on common pathways of neuronal injury. These include lysosomal dysfunction, mitochondrial impairment, axonal transport defects, neuroinflammation, and, in selected disorders, peroxisomal dysfunction with toxic lipid accumulation. Together, these processes disrupt cellular homeostasis, impair synaptic and metabolic integrity, and ultimately promote progressive neurodegeneration.

**Figure 2:**
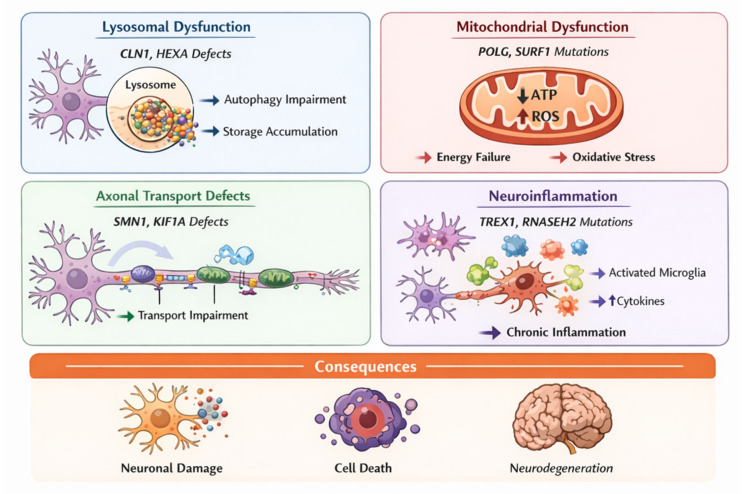
Major molecular mechanisms contributing to pediatric neurodegenerative diseases and their convergence on neuronal injury.

Figure 2 summarizes the principal molecular mechanisms involved in pediatric neurodegenerative diseases and illustrates how distinct cellular disturbances converge toward neuronal injury and neurodegeneration. Lysosomal dysfunction, exemplified by defects in genes such as CLN1 and HEXA, leads to impaired substrate degradation, defective autophagy, and intracellular accumulation of storage material. Mitochondrial dysfunction, associated with pathogenic variants in genes involved in mitochondrial function such as POLG (DNA polymerase gamma, catalytic subunit) and SURF1 (SURF1 cytochrome c oxidase assembly factor), results in impaired oxidative phosphorylation, reduced ATP production, and increased oxidative stress. Axonal transport defects, linked to genes such as SMN1 (survival motor neuron 1) and KIF1A (kinesin family member 1A), compromise intracellular trafficking required for synaptic maintenance and neuronal survival. Neuroinflammatory mechanisms, associated with genes such as TREX1 (three-prime repair exonuclease 1) and RNASEH2 (ribonuclease H2), promote microglial activation and chronic inflammatory signaling, further exacerbating neuronal damage. In selected disorders, peroxisomal dysfunction contributes through impaired VLCFA metabolism and toxic lipid accumulation, with secondary effects on myelin stability and axonal integrity. Despite arising from different molecular pathways, these mechanisms share common downstream consequences, including synaptic dysfunction, neuronal injury, impaired brain maturation, and progressive neurodegeneration. In children, these effects may also disrupt ongoing brain development, including synaptogenesis, myelination, and circuit maturation, thereby contributing not only to developmental regression but also to developmental delay and failure of expected neurologic acquisition.

**Figure 3:**
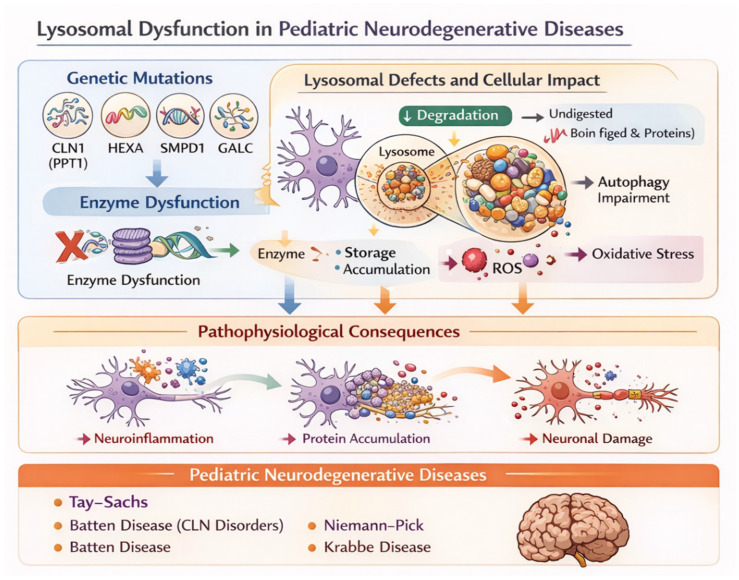
Mechanistic Overview of Lysosomal Dysfunction in Pediatric Neurodegenerative Diseases. **Legend:** *CLN1 (PPT1)*—ceroid lipofuscinosis neuronal 1; *HEXA*—hexosaminidase A; *SMPD1*—sphingomyelin phosphodiesterase 1; *GALC*—galactosylceramidase.

Figure 3 illustrates the molecular basis and downstream consequences of lysosomal dysfunction in pediatric neurodegenerative diseases, highlighting how pathogenic variants lead to enzyme deficiency, impaired lysosomal function, and progressive neuronal injury.

At the genetic level, pathogenic variants in lysosomal genes associated genes such as *CLN1 (PPT1)*, *HEXA*, *SMPD1*, and *GALC* are highlighted. These genes encode enzymes essential for lysosomal degradation pathways. Pathogenic variants lead to enzyme dysfunction, which compromises the lysosome’s capacity to degrade macromolecules, including lipids and proteins.

As a consequence of reduced lysosomal degradation, undegraded substrates accumulate within the lysosomal compartment, resulting in intracellular storage material buildup. This accumulation disrupts cellular homeostasis and impairs autophagy, a critical pathway responsible for the turnover of damaged organelles and aggregated proteins. Autophagy impairment further exacerbates substrate accumulation, establishing a pathogenic feedback loop. In parallel, defective lysosomal function contributes to increased ROS production and oxidative stress, amplifying cellular damage.

These molecular alterations culminate in major pathophysiological consequences, including neuroinflammation, progressive protein accumulation, and neuronal damage. Persistent storage burden and oxidative stress activate inflammatory pathways and glial responses, which further accelerate neuronal dysfunction.

Clinically, these mechanisms underlie several pediatric lysosomal storage and neurodegenerative disorders, including Tay–Sachs disease, Batten disease (neuronal ceroid lipofuscinoses), Niemann–Pick disease, and Krabbe disease [19,20,21,22,23,48,78]. Despite genetic heterogeneity, these conditions share a common pathogenic cascade characterized by lysosomal failure, substrate accumulation, neuroinflammation, and progressive neurodegeneration.

Overall, the diagram underscores how primary lysosomal enzyme defects initiate a cascade of cellular disturbances that converge on neuronal injury, providing a mechanistic framework for understanding pediatric lysosomal neurodegenerative diseases.

Figure 3 illustrates how pathogenic variants in lysosomal genes (e.g., CLN1/PPT1, HEXA, SMPD1, and GALC) lead to enzymatic deficiencies that impair lysosomal degradation. Accumulation of undegraded substrates disrupts autophagic flux and cellular clearance, promoting lysosomal stress and secondary mitochondrial dysfunction. Increased generation of reactive oxygen species (ROS) and oxidative stress further activate neuroinflammatory pathways and promote protein aggregation. Together, these convergent mechanisms lead to synaptic dysfunction and progressive neuronal injury, thereby underlying the neurodegenerative phenotype observed in pediatric lysosomal storage disorders [97,98,99,100,101,102,103,104].

## 6. Diagnosis in Pediatric Neurodegenerative Diseases

Pediatric neurodegenerative diseases, unlike adult-onset neurodegenerative diseases, frequently present with developmental delays, regression of previously acquired milestones, epilepsy, movement disorders, and multisystem involvement. Early and accurate diagnosis is essential for prognosis, genetic counseling, therapeutic interventions (including enzyme replacement and gene therapy), and inclusion in clinical trials [105,106,107].

Recent advances in next-generation sequencing (NGS), metabolomics, and neuroimaging have significantly improved diagnostic yield, shifting the paradigm from phenotype-based to genotype-based approaches [108,109,110].

However, the complexity of establishing a diagnosis remains high due to phenotypic overlap, rarity of individual disorders, and variable expressivity.

### 6.1. Clinical and Neuroimaging Evaluation

Developmental regression, progressive motor decline, epilepsy, and multisystem involvement are major diagnostic red flags. Recognition of the brain MRI pattern (white matter changes, basal ganglia abnormalities, cerebellar atrophy) directs targeted metabolic and genetic investigations.

#### 6.1.1. Clinical Assessment

Clinical warning signs suggestive of neurodegeneration rather than static encephalopathy include developmental regression after a period of previously acquired skills, progressive cognitive decline, loss of motor abilities, movement disorders (including dystonia and ataxia), refractory seizures, visual or hearing impairment, and multisystem involvement such as hepatosplenomegaly or cardiomyopathy.

Developmental regression is an important clinical warning sign, but it is not specific to pediatric neurodegenerative disease and may also occur in epileptic encephalopathies, autoimmune encephalitis, Rett syndrome, and other non-degenerative conditions. In pediatric neurodegeneration, regression is more suggestive when it is progressive, accompanied by additional neurological or multisystem red flags, and supported by characteristic neuroimaging, biochemical, or genetic findings.

In addition to careful neurological examination and developmental history, detailed family history is a pivotal component of the initial clinical assessment. Particular attention should be paid to consanguinity, similarly affected siblings, recurrent infant deaths, unexplained epilepsy, movement disorders, psychiatric disease, dementia, or progressive neurological decline in relatives, as these features may provide important clues regarding inheritance pattern and disease category. Family history is relevant not only for classic Mendelian presentation, but also because emerging evidence suggests that heterozygous variants in genes usually associated with recessive disorders may, in selected cases, contribute to neurological phenotypes or act as disease-risk modifiers. This has been discussed in relation to NPC1, for which subclinical cognitive and neurophysiological abnormalities in heterozygous carriers and rare late-onset familial neurodegenerative presentations have been reported. Therefore, familial anamnesis should be integrated systematically into the diagnostic workup, even when the suspected disorder is classically considered autosomal recessive [105,111,112,113,114].

#### 6.1.2. Neuroimaging

Magnetic resonance imaging (MRI) remains essential for diagnosis. Characteristic patterns can strongly suggest specific disorders. For example: the presence of symmetrical white matter abnormalities suggests leukodystrophies; basal ganglia involvement is a common finding in mitochondrial disorders, NBIA. Cerebellar atrophy occurs in spinocerebellar ataxias, mitochondrial diseases. Diffusion restriction in acute metabolic crises. MRI pattern recognition algorithms significantly improve targeted genetic testing [42,115,116,117].

### 6.2. Laboratory and Metabolic Tests

The initial laboratory evaluation will include plasma amino acids, acylcarnitine profile, lactate and pyruvate, VLCFA, and urinary organic acids. When necessary, enzymatic tests will be performed. Biochemical screening plays a critical role in identifying treatable inborn errors of metabolism, including urea cycle disorders, biotinidase deficiency, and selected LSDs. However, normal metabolic screening does not exclude genetic neurodegenerative diseases [118,119,120].

### 6.3. Genetic Testing

Genetic testing, including whole-exome sequencing (WES) and whole-genome sequencing (WGS), is increasingly used as a first-line tool for establishing a molecular diagnosis in children with unexplained neurodevelopmental or neurodegenerative disorders. In suspected mitochondrial disease, the diagnostic strategy requires particular attention because pathogenic variants may involve either mtDNA or nuclear genes encoding mitochondrial proteins. Accordingly, currently available genetic approaches include full mtDNA sequencing, targeted nuclear mitochondrial gene panels, combined dual genomic testing assessing both mtDNA and nuclear genes, as well as WES/WGS for unresolved or genetically heterogeneous phenotypes. Current recommendations favor next-generation sequencing of the complete mtDNA genome rather than limited testing for a small number of common mtDNA variants, while also recognizing that heteroplasmy levels may differ across tissues; therefore, analysis of blood alone may be insufficient in selected cases, and testing of other tissues may be necessary when clinical suspicion remains high. Targeted gene panels may be useful when the phenotype strongly suggests a mitochondrial disorder, whereas WES or WGS are particularly valuable in atypical presentations, in patients with broad multisystem involvement, or when prior targeted testing is non-diagnostic. Diagnostic interpretation is further strengthened by **trio-based analysis**, integration with biochemical and neuroimaging findings, and careful consideration of inheritance pattern and tissue specificity [121,122,123].

### 6.4. Emerging Diagnostic Approaches

New or emerging medical technologies or methods are being used to detect and monitor diseases more accurately, earlier, and often less invasively than traditional methods. Emerging diagnostic approaches include metabolomics and lipidomics to identify disease-specific metabolic signatures; transcriptomic profiling to assess gene-expression abnormalities; RNA sequencing with dedicated splicing or isoform analysis to detect transcript-level defects; long-read sequencing for structural variants and repetitive expansions; and machine learning-assisted MRI analysis. The integration of multi-omics approaches is increasingly recommended for complex undiagnosed cases.

Diagnosis of pediatric neurodegenerative diseases requires a systematic, multidisciplinary approach integrating clinical phenotyping, neuroimaging, biochemical testing, and genomic technologies. A stepwise but increasingly genome-first strategy is emerging as the standard of care. Continued integration of multi-omics and AI-assisted imaging will likely further improve diagnostic precision and enable earlier therapeutic intervention [124,125,126].

Pediatric neurodegenerative diseases typically present with developmental regression (loss of previously acquired skills) and/or progressive neurologic deterioration. The clinical phenotype often includes new-onset or worsening epilepsy, movement disorders (dystonia, parkinsonism, ataxia), spasticity, progressive cognitive decline, and bulbar/brainstem signs (dysphagia, oculomotor abnormalities). Systemic “clues” can narrow the differential: hepatosplenomegaly and dysostosis suggest lysosomal storage disorders, adrenal insufficiency may indicate peroxisomal disease (e.g., X-ALD), and exercise intolerance, failure to thrive, or episodic decompensation with lactic acidosis can point toward mitochondrial disease [124,127,128].

This algorithm emphasizes the central role of genomic technologies in accelerating etiological diagnosis, enabling genotype–phenotype correlations, informing precision therapies, and supporting genetic counseling. Overall, the figure highlights a logical, effective, and evidence-based pathway designed to reduce diagnostic delay and optimize patient outcomes.

**Figure 4:**
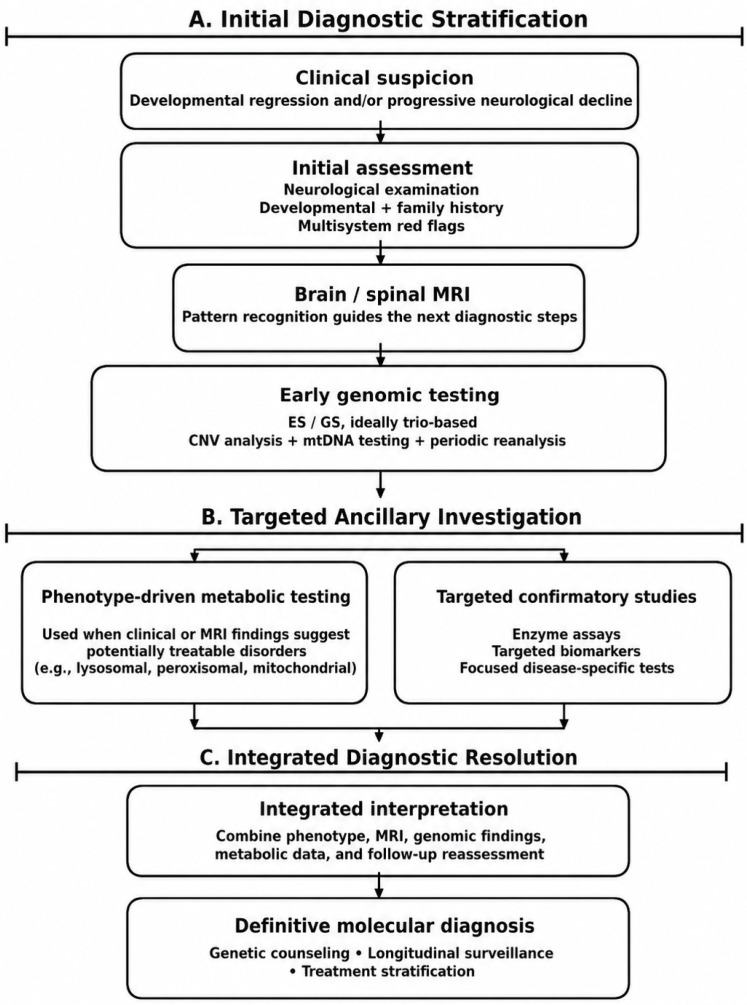
Proposed Diagnostic Workflow for Children with Developmental Regression and/or Progressive Neurological Decline. **Legend:** A = Initial Diagnostic Stratification; B = Targeted Ancillary Investigation; C = Integrated Diagnostic Resolution; MRI, magnetic resonance imaging; ES, exome sequencing; GS, genome sequencing; CNV, copy number variation; mtDNA, mitochondrial DNA.

The proposed diagnostic workflow for children with developmental regression and/or progressive neurological decline is shown in Figure 4. The algorithm integrates initial clinical assessment, brain/spinal MRI, early genomic testing, phenotype-driven metabolic investigations, and targeted confirmatory studies to support integrated interpretation and establish a definitive molecular diagnosis. It emphasizes the early use of exome or genome sequencing, ideally trio-based, in combination with CNV analysis, mtDNA testing, and periodic reanalysis, while metabolic and disease-specific investigations are guided by the clinical and neuroimaging phenotype, particularly when potentially treatable disorders are suspected. This integrated approach informs genetic counseling, longitudinal surveillance, and treatment stratification.

Table 6 highlights the complementary roles of neuroimaging, metabolic and enzymatic testing, targeted gene panels, whole-exome/genome sequencing, and emerging multi-omics approaches in establishing an etiological diagnosis while acknowledging constraints in sensitivity, specificity, accessibility, and interpretative complexity [125,126,129,130].

Diagnosis is optimized by combining (I) careful phenotyping and timeline of regression, (II) brain MRI (often pattern-defining in leukodystrophies), (III) targeted biochemical screening when clinically indicated (e.g., very-long-chain fatty acids; lysosomal enzyme assays), and (IV) early genetic testing (chromosomal microarray and/or exome/genome sequencing), which frequently yields the highest diagnostic return in unexplained neurodevelopmental disorders and progressive regression syndromes [126,131,132,133].

Early diagnosis is particularly important given the increasing availability of disease-modifying therapies, such as gene therapy for spinal muscular atrophy and enzyme replacement for lysosomal storage disorders.

However, challenges in diagnosis include phenotypic heterogeneity, overlapping imaging findings, variants of uncertain significance (VUS), limited access to advanced testing, and ethical considerations in genomic testing.

Newborn screening and presymptomatic management are becoming increasingly important in selected pediatric neurodegenerative disorders, but their clinical implications differ across disease categories. In disorders such as X-linked adrenoleukodystrophy (X-ALD), newborn screening has an established framework that enables identification of at-risk individuals before neurological symptoms develop. However, early detection does not imply immediate treatment in all screen-positive children. Rather, it requires structured longitudinal surveillance, including serial brain MRI during the age window of highest risk for cerebral conversion, together with endocrine monitoring for adrenal insufficiency, in order to identify the optimal moment for therapeutic intervention. By contrast, in MLD, the value of early diagnosis is closely linked to the fact that disease-modifying therapies are most effective when administered presymptomatically or at a very early symptomatic stage, before substantial and irreversible neurological injury has occurred. These distinctions illustrate that newborn screening should not be viewed simply as an earlier diagnostic tool, but as part of a broader clinical framework in which surveillance strategy, phenotype-specific risk, and treatment timing are tightly interconnected. Accordingly, the central clinical principle is not only early detection, but also the ability to match diagnosis with the appropriate monitoring pathway and the correct therapeutic window [134].

## 7. Management Strategies in Pediatric Neurodegenerative Disorders

### 7.1. A Stratified Therapeutic Framework for Pediatric Neurodegenerative Disorders

The management of pediatric neurodegenerative disorders has undergone a substantial conceptual shift over the last decade. Historically, treatment was predominantly supportive and focused on seizure control, nutritional support, respiratory care, rehabilitation, and palliation. This approach reflected the reality that most childhood neurodegenerative diseases were diagnosed late, understood incompletely, and lacked disease-modifying options. Advances in molecular genetics, newborn screening, neuroimaging, and translational therapeutics have begun to alter this landscape. For a growing but still limited subset of conditions, treatment now extends beyond symptomatic control toward mechanism-based intervention. Nevertheless, this transition should not be overstated: pediatric neurodegeneration is not yet a uniformly treatable field, and therapeutic progress remains highly uneven across disorders [32,34,66,121,122,123].

A critical distinction must therefore be made between supportive care, established disease-modifying therapies used in selected indications, and emerging or experimental precision-based interventions [135,136,137,138,139,140,141,142,143,144,145,146,147,148,149,150,151,152,153].

These approaches are often discussed together under the broad umbrella of targeted therapy, yet they differ profoundly in biological rationale, clinical maturity, regulatory status, route of administration, evidence base, and disease specificity. Enzyme replacement therapy, hematopoietic stem cell transplantation, gene therapy, antisense-mediated RNA modulation, and genome editing should not be viewed as interchangeable expressions of one therapeutic paradigm. Rather, they represent distinct platforms with different strengths, different limitations, and very different levels of readiness for routine clinical application.

At present, only a relatively small number of pediatric neurodegenerative disorders benefit from therapies with clearly demonstrated disease-modifying effects in clinical practice. Even in these conditions, however, treatment efficacy is usually constrained by the timing of intervention. A central principle across the field is that molecular correction does not necessarily reverse established neural circuit injury. Once significant neuronal loss, axonal degeneration, or white-matter destruction has occurred, subsequent treatment may stabilize disease or slow further progression without restoring normal neurological development. This is especially important in children, in whom the nervous system is still undergoing synaptogenesis, myelination, and network refinement. In this setting, delayed treatment may fail not only because degeneration is advanced, but also because abnormal development and degeneration have already become biologically intertwined. Accordingly, the therapeutic window in many pediatric neurodegenerative disorders is narrow, and the greatest benefit is often observed in presymptomatic or very early symptomatic stages [134,137,138,139,140,141,142,143,144,145,146].

Another major limitation is delivery to the central nervous system. Many of the molecules used in advanced therapies, including recombinant enzymes, viral vectors, and nucleic acid–based agents, do not readily cross the blood–brain barrier in therapeutically sufficient concentrations. As a result, route of administration becomes a decisive determinant of efficacy. Intravenous delivery may be effective for systemic manifestations yet inadequate for progressive neurological disease, whereas intrathecal, intracerebroventricular, or otherwise CNS-directed strategies may be necessary to achieve biologically meaningful benefit. This issue is particularly relevant for lysosomal and other metabolic disorders with prominent brain involvement, and it partly explains why some therapies produce clear peripheral improvement but limited central neurological rescue [135,136,137,138,147,148,149,150,151,152,153,154,155,156].

Clinical response is also markedly heterogeneous. Therapeutic outcomes vary according to genotype, residual enzyme or protein activity, inflammatory burden, disease stage, tissue distribution, age at treatment, and baseline neurological reserve. Disorders grouped under the same broad mechanistic class may differ substantially in reversibility, rate of progression, and treatment susceptibility. Even within a single disease, phenotypic spectrum can be wide, and genotype–phenotype correlations may alter both prognosis and therapeutic expectations. For this reason, management strategies should not be generalized too broadly across disease categories, and caution is needed when extrapolating success from one disorder or one therapeutic platform to another.

In addition, many advanced therapies are associated with major **practical and ethical constraints**. Hematopoietic stem cell–based strategies may require myeloablative conditioning and are therefore linked to procedure-related toxicity. Viral gene therapies raise issues of immune response, vector biodistribution, transgene durability, and long-term safety monitoring. RNA-based and individualized molecular therapies may require repeated administration and highly specialized infrastructure. Genome editing approaches remain largely investigational and introduce further concerns related to off-target modification, permanence of genomic alteration, tissue specificity, and ethical governance. Even when therapeutic efficacy is promising, the real-world implementation of these interventions is often restricted by cost, limited availability, regulatory heterogeneity, and profound inequalities in access between healthcare systems.

These considerations highlight an important tension within the field. On one hand, pediatric neurodegenerative disease has become one of the clearest examples of the promise of precision medicine. On the other hand, the field remains characterized by a pronounced gap between biological plausibility and broad clinical applicability. For some disorders, targeted intervention has already transformed prognosis when introduced sufficiently early. For many others, however, treatment remains supportive, experimental, or accessible only in restricted contexts. The therapeutic literature must therefore be interpreted with balance: neither therapeutic nihilism nor excessive optimism adequately reflects current reality.

Against this background, the management of pediatric neurodegenerative disorders is best understood as a stratified framework. The first level consists of comprehensive multidisciplinary supportive care, which remains essential for all patients regardless of whether a disease-modifying therapy exists. The second level includes established disease-modifying approaches with demonstrated clinical value in selected disorders, such as enzyme replacement therapy, hematopoietic stem cell transplantation, or approved gene-based treatments. The third level includes emerging precision platforms—such as ex vivo lentiviral hematopoietic stem cell gene therapy, in vivo adeno-associated virus–based gene transfer, antisense oligonucleotide strategies, and other transcript-level interventions—that are clinically advanced in some indications but not broadly established across the field. The fourth level encompasses experimental and investigational approaches, including genome editing and individualized mutation-specific therapeutics, which remain promising but are not yet standard care for most pediatric neurodegenerative conditions.

Accordingly, the sections that follow discuss management not as a single therapeutic narrative, but as a spectrum ranging from supportive stabilization to highly specific molecular intervention. This structure is necessary in order to reflect the major differences in evidence level, disease applicability, timing sensitivity, and translational maturity across currently available strategies. It also emphasizes a key clinical reality: the success of pediatric neurodegenerative management depends not only on therapeutic innovation itself, but equally on early diagnosis, accurate molecular classification, careful patient selection, longitudinal monitoring, and equitable access to specialized multidisciplinary care [121,122,123,157,158,159,160,161,162,163,164,165,166,167,168,169,170,171,172].

The following sections review current management strategies according to their level of clinical maturity, mechanistic rationale, and relevance to pediatric neurodegenerative disorders.

#### 7.1.1. Enzyme Replacement Therapy

Enzyme replacement therapy (ERT) is an etiology-directed approach designed to restore deficient lysosomal or metabolic enzyme activity through the administration of recombinant protein. In pediatric neurodegenerative disorders, ERT is most relevant in diseases in which the missing enzyme is well characterized and can be replaced in a biologically meaningful manner. However, a major limitation of conventional intravenous ERT is its poor penetration across the blood–brain barrier, which restricts efficacy in disorders with substantial central nervous system involvement. For this reason, direct CNS delivery strategies have become particularly important in neuronopathic lysosomal diseases. A representative example is CLN2 disease, in which cerliponase alfa, a recombinant form of tripeptidyl peptidase 1, is administered intracerebroventricularly in order to bypass the blood–brain barrier and directly target the brain. This approach has been shown to slow the decline of motor-language function, including in long-term follow-up studies. These observations illustrate that ERT should not be considered a uniform platform, because therapeutic efficacy depends not only on the enzyme replaced, but also on the route of administration and the capacity to reach the relevant tissue compartment. In clinical practice, the greatest benefit is generally achieved when treatment is initiated early, before advanced and irreversible neurological injury has occurred [135,136,137,138].

For clarity, Table 7 summarizes representative disease-specific precision therapies in pediatric neurodegenerative disorders, highlighting the disorder, main pathogenic pathway, therapeutic modality, delivery platform, representative example, and the optimal timing of intervention.

#### 7.1.2. Ex Vivo Lentiviral Hematopoietic Stem Cell Gene Therapy

Ex vivo lentiviral hematopoietic stem cell gene therapy represents a conceptually distinct disease-modifying strategy. In this platform, autologous hematopoietic stem cells are collected from the patient, transduced ex vivo with a lentiviral vector carrying a functional copy of the pathogenic variant, and then reinfused after conditioning. The therapeutic rationale is that engrafted myeloid-lineage cells can subsequently contribute to enzyme delivery or metabolic correction within the central nervous system. This strategy has shown major clinical value in metachromatic leukodystrophy (MLD), where atidarsagene autotemcel has demonstrated benefit particularly in presymptomatic late-infantile and early symptomatic early-juvenile patients, emphasizing the importance of treatment before rapid neurological deterioration is established. A related approach has also been applied in early cerebral X-linked adrenoleukodystrophy, in which autologous CD34+ cells are genetically corrected ex vivo and reinfused to modify the inflammatory demyelinating process. In both disorders, efficacy is strongly stage dependent, and outcomes are most favorable when treatment is delivered during an early disease window, prior to severe functional decline. Thus, ex vivo lentiviral HSC gene therapy differs fundamentally from direct in vivo gene transfer, because it relies on cellular engraftment and secondary CNS benefit mediated by hematopoietic-derived cells [143,144,145,146,147].

#### 7.1.3. In Vivo AAV-Based Gene Therapy

In vivo AAV-based gene therapy involves direct administration of a viral vector carrying a therapeutic gene to the patient, without ex vivo cell manipulation. This platform is designed to achieve sustained expression of the missing or pathogenic variant in target tissues and has become a major advance in selected pediatric monogenic neurological disorders. A key example is spinal muscular atrophy (SMA), in which onasemnogene abeparvovec uses a systemically delivered AAV9 vector to provide a functional copy of SMN1. Because SMA is characterized by progressive loss of motor neurons, the timing of intervention is critical: the greatest clinical benefit is observed when treatment is administered before substantial motor neuron degeneration, ideally during a presymptomatic or very early symptomatic stage. Compared with ex vivo lentiviral strategies, in vivo AAV-based therapy does not depend on stem-cell collection or engraftment; instead, its success depends on vector biodistribution, tissue tropism, immune considerations, and sufficient transgene expression in the relevant target cells. For these reasons, AAV-based therapy should be discussed separately from hematopoietic stem cell gene therapy, even though both fall under the broader category of gene-based treatment [148,149].

#### 7.1.4. RNA-Based and Molecular Therapies

RNA-based therapeutics constitute a mechanistically distinct class of precision interventions that act at the level of RNA processing or transcript abundance rather than by direct gene replacement. Among these, antisense oligonucleotides (ASOs) are the most clinically advanced platform and can be designed to modify pre-mRNA splicing, promote degradation of toxic transcripts, or reduce expression of pathogenic alleles in a sequence-specific manner. Their therapeutic logic is therefore different from both enzyme replacement and viral gene transfer, because they do not introduce a permanent gene copy but instead modulate downstream RNA behavior. The best-established pediatric example is nusinersen in spinal muscular atrophy, which enhances inclusion of exon 7 in SMN2 transcripts and thereby increases production of functional SMN protein. More broadly, ASO-based strategies are being explored for a growing number of monogenic neurological disorders, including ultra-rare individualized applications, particularly when the disease mechanism involves aberrant splicing, toxic gain-of-function RNA species, or a transcript amenable to selective suppression. However, these therapies also present important limitations, including the need for repeated administration, challenges of CNS distribution, variable durability of effect, and the complexity of mutation-specific development. Small-molecule chaperones, nonsense read-through compounds, and related molecular approaches may also provide mutation- or pathway-specific benefit in selected disorders, but their applicability remains highly disease dependent. Overall, RNA-based therapies are best viewed as a separate therapeutic platform within pediatric neurodegeneration, particularly relevant for disorders in which transcript-level correction is more feasible than full gene replacement [150,151,152,153].

#### 7.1.5. Emerging Genome Editing Approaches

Genome editing approaches, particularly CRISPR-based systems, are being actively investigated as a potential next generation of precision therapies for pediatric neurological and neurodegenerative disorders. Unlike conventional gene replacement, genome editing seeks to directly modify the endogenous genomic sequence or regulate native gene expression, offering the theoretical possibility of durable or even permanent correction of pathogenic variants. Depending on the platform, these strategies may be used to disrupt harmful alleles, repair point mutations, insert corrective sequences, or modulate gene expression through base editing, prime editing, or CRISPR-associated RNA-targeting technologies. This conceptual flexibility makes genome editing especially attractive for monogenic disorders in which the causative mutation is well defined. Nevertheless, in pediatric neurodegeneration, these approaches remain largely experimental, and major translational challenges persist, including delivery to the central nervous system, cell-type specificity, off-target editing, long-term safety, immunogenicity, and ethical considerations related to irreversible genomic modification in developing tissues. At present, CRISPR-based strategies should therefore be regarded as promising but investigational tools whose future clinical role will depend on continued advances in vector engineering, editing precision, and disease-specific preclinical validation [149,152,154,155,156,157].

Beyond currently established platforms, the therapeutic landscape is continuing to expand toward individualized precision approaches, including mutation-specific antisense oligonucleotides and other patient-tailored interventions for ultra-rare neurogenetic disorders, although these strategies remain available only in highly selected settings at present [158].

### 7.2. Symptomatic and Multidisciplinary Supportive Care

Despite therapeutic advances, many pediatric neurodegenerative disorders remain without disease-modifying treatment. Therefore, multidisciplinary care is essential and includes seizure management with personalized anticonvulsant medications; treatment for spasticity (oral baclofen, intrathecal baclofen pumps, botulinum toxin); nutritional optimization and gastrostomy when necessary; respiratory monitoring and non-invasive ventilation; physical, occupational, and speech therapy; neuropsychological and behavioral support; and integration of early palliative care in progressive conditions. Longitudinal surveillance with standardized neurological scales and biomarker monitoring is essential to assess disease trajectory and therapeutic response [159,160,161].

### 7.3. Precision Medicine and Multi-Omics Integration

The integration of genomics, transcriptomics, proteomics, and metabolomics (multi-omics) has improved diagnostic yield and mechanistic understanding of pediatric neurodegeneration. Molecular stratification facilitates genotype–phenotype correlation; biomarker discovery; identification of therapeutic targets; and enrollment in genotype-specific clinical trials.

Whole exome sequencing and WGS have significantly shortened diagnostic odysseys, while functional validation using transcriptomic and proteomic profiling helps resolve variants of uncertain significance. Future management strategies will likely combine gene replacement, gene editing, RNA modulation, and pharmacological interventions within individualized treatment settings [121,123,162,163,164,165,166,167].

Emerging fluid biomarkers, particularly neurofilament light chain (NfL), are increasingly relevant in pediatric neurodegeneration as indicators of neuroaxonal injury and therapeutic response. Disease-specific studies in cerebral X-linked adrenoleukodystrophy and CLN2 disease support the utility of NfL for monitoring disease activity and treatment-related change, highlighting its growing translational value in both clinical care and trial design [168].

### 7.4. Ethical and Psychosocial Considerations

The diagnosis of a neurodegenerative disease in a child has profound ethical and psychosocial implications. Families face complex decisions regarding genetic testing, reproductive choices, and aggressive interventions. Psychological support, palliative care, and clear communication are essential components of comprehensive care [169,170,171,172].

**Figure 5:**
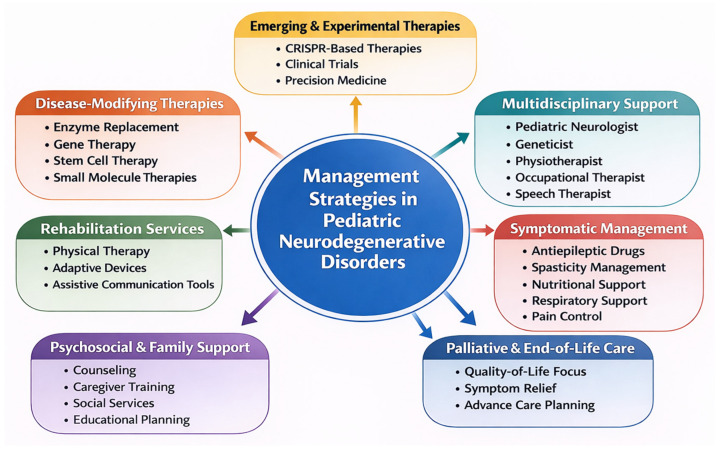
Management Strategies for Pediatric Neurodegenerative Disorders.

Figure 5 presents a conceptual framework for the management of pediatric neurodegenerative disorders using a central hub-and-spoke design. At the center is the core theme, “Management Strategies in Pediatric Neurodegenerative Disorders.” Surrounding this are seven major management domains: disease-modifying therapies, symptomatic management, multidisciplinary support, rehabilitation services, psychosocial and family support, palliative and end-of-life care, and emerging/experimental therapies.

Each domain includes key examples of interventions, illustrating that care extends beyond pharmacologic treatment to encompass rehabilitation, supportive services, and family-centered care. The directional arrows emphasize the integrated and interconnected nature of these strategies.

The note at the bottom highlights that effective management requires a personalized, multidisciplinary approach tailored to each child’s specific needs.

## 8. Limitations

This review has several limitations. First, it was designed as a narrative comprehensive review rather than a formal systematic review or meta-analysis; consequently, the literature selection process, although structured, may remain subject to selection bias. Second, the field of pediatric neurodegenerative diseases is highly heterogeneous, encompassing numerous ultra-rare disorders with variable phenotypes, limited natu-ral-history data, and uneven representation in the published literature. As a result, the strength of evidence differs substantially across disease groups.

Third, much of the currently available evidence is derived from observational studies, expert consensus documents, and disease-specific cohorts rather than randomized controlled trials, particularly for rare pediatric conditions. Fourth, rapidly evolving areas such as genomic diagnostics, newborn screening, gene therapy, and RNA-based therapeutics may change substantially over time, which can affect the long-term currency of specific statements. Finally, because the aim of this review was to provide a broad integrative synthesis, some highly specialized disorders and emerging molecular mechanisms may not have been discussed in the same level of detail as more extensively studied conditions.

Despite these limitations, we believe this review provides a clinically relevant and mechanistically coherent overview of pediatric neurodegenerative diseases, with emphasis on shared pathogenic pathways, diagnostic strategies, and evolving therapeutic perspectives.

## 9. Conclusions and Future Directions

The management of pediatric neurodegenerative disorders is undergoing a paradigm shift from predominantly supportive care to molecularly targeted precision medicine.

Advances in next-generation sequencing and multi-omics technologies have substantially improved diagnostic yield, enabling earlier identification of actionable genetic etiologies.

The emergence of gene therapy, RNA-based therapeutics, and enzyme replacement strategies has demonstrated that timely intervention can significantly alter disease trajectories in selected disorders.

Nevertheless, substantial challenges remain, including limited central nervous system delivery, immune responses to viral vectors, high treatment costs, and inequitable global access to advanced therapeutics.

Furthermore, many ultra-rare conditions still lack disease-modifying options, underscoring the need for collaborative translational research and international registries.

Future therapeutic paradigms will likely integrate genome editing, biomarker-driven stratification, and individualized treatment algorithms within multidisciplinary care frameworks.

Continued investment in precision neurology holds promise for transforming pediatric neurodegenerative diseases from relentlessly progressive conditions into manageable or potentially reversible disorders.

## Figures and Tables

**Table 1:** **Conceptual Framework for the Classification of Neurodegenerative Diseases in Children.**

Classification Domain	Definition	Examples	Comment
**Etiologic–molecular categories**	Disorders grouped by the principal defective organelle, pathway, or cellular system	Lysosomal, mitochondrial, peroxisomal, myelin-related/glial disorders	Forms the primary classification framework used in this review
**Clinico-radiologic syndromes**	Disorders grouped by shared clinical or MRI phenotype	Leukodystrophies, developmental regression syndromes, epileptic neurodegenerative encephalopathies	Useful in practice, but not mechanistically uniform
**Cross-cutting mechanistic pathways**	Shared pathogenic processes operating across multiple disease classes	Autophagy–lysosome dysfunction, neuroinflammation, oxidative stress, axonal transport failure	Helps explain phenotypic overlap and therapeutic convergence
**Genetic architecture groupings**	Disorders grouped by mutational mechanism rather than phenotype or organelle	Repeat expansion disorders, selected DNA repair disorders	Important biologically, but not equivalent to top-level etiologic classes

**Table 2:** Molecular, Genetic, and Clinical Features of Selected Pediatric Lysosomal Storage Disorders.

No.	Disorder	Enzyme Deficiency	Gene Involved	Inheritance Pattern	Main Pathophysiology	Common Clinical Features (Children)
1	Gaucher Disease	Glucocerebrosidase	*GBA1*	Autosomal recessive	Accumulation of glucocerebroside in macrophages (Gaucher cells)	Hepatosplenomegaly, anemia, thrombocytopenia, bone pain/crises, fatigue
2	Tay–Sachs Disease	Hexosaminidase A	*HEXA*	Autosomal recessive	GM2 ganglioside accumulation in neurons	Progressive neurodegeneration, developmental regression, seizures, vision loss, early death (infantile form)
3	Niemann–Pick Disease	Acid sphingomyelinase deficiency, types A/B; or impaired intracellular cholesterol trafficking, types C1/C2	*SMPD1*, types A/B; *NPC1*, type C1 gene; or *NPC2*, type C2 gene	Autosomal recessive	Sphingomyelin accumulation in lysosomes, types A/B; or unesterified cholesterol and glycosphingolipid accumulation in lysosomes/late endosomes, types C1/C2	Developmental delay, hepatosplenomegaly, neurological decline, ataxia
4	Pompe Disease	Acid α-glucosidase (GAA)	*GAA*	Autosomal recessive	Lysosomal glycogen accumulation (especially in muscle cells)	Hypotonia, muscle weakness, hypertrophic cardiomyopathy (infantile form), respiratory insufficiency
5	Hurler Syndrome (MPS I)	α-L-iduronidase	*IDUA*	Autosomal recessive	Accumulation of glycosaminoglycans (dermatan sulfate and heparan sulfate)	Coarse facial features, dysostosis multiplex, developmental delay, organomegaly

**Legend:** *GAA*—Acid α-glucosidase; *GBA1*—Glucosylceramidase beta 1 gene; *GM2*—Monosialotetrahexosylganglioside; *HEXA*—hexosaminidase subunit alpha gene; *IDUA*—Alpha-L-iduronidase gene; LSD—Lysosomal storage disorder; MPS I—Mucopolysaccharidosis type I; NPC1—Niemann–Pick disease, type *C1* gene; NPC2—Niemann–Pick disease, type *C2* gene; *SMPD1*—Sphingomyelin phosphodiesterase 1 gene.

**Table 3:** Selected inherited white matter disorders: genetic basis, principal pathobiological mechanisms, characteristic pediatric manifestations, and disease-modifying or emerging therapeutic approaches.

Disorder	Inheritance	Causative Gene(s) *	Principal Pathobiological Mechanism	Characteristic Pediatric Manifestations	Disease-Modifying/Emerging Therapies **
**Krabbe disease (GLD)**	Autosomal recessive	*GALC*	*GALC* deficiency with psychosine accumulation, leading to oligodendrocyte injury, globoid macrophage formation, severe neuroinflammation, and central/peripheral demyelination	Irritability, hypertonia/spasticity, developmental regression, peripheral neuropathy, optic atrophy	Early HSCT in presymptomatic or very early symptomatic infants; investigational gene therapy and adjunct anti-inflammatory approaches
**Metachromatic leukodystrophy (MLD)**	Autosomal recessive	*ARSA*; rarely *PSAP* (saposin B deficiency)	Impaired sulfatide degradation, resulting in sulfatide accumulation in the CNS and PNS, progressive demyelination, and axonal loss	Gait disturbance, hypotonia progressing to spasticity/ataxia, cognitive decline, peripheral neuropathy	Ex vivo lentiviral gene therapy; HSCT in selected presymptomatic or early-stage cases; investigational enzyme replacement strategies
**X-linked adrenoleukodystrophy (X-ALD)**	X-linked recessive	*ABCD1*	Defective peroxisomal transport of very-long-chain fatty acids (VLCFAs), causing VLCFA accumulation, inflammatory cerebral demyelination, axonopathy, and adrenocortical dysfunction	Behavioral and cognitive decline, visual and auditory deficits, rapid neurologic deterioration in childhood cerebral ALD; adrenal insufficiency	HSCT for early cerebral disease; ex vivo lentiviral gene therapy in eligible patients; glucocorticoid replacement for adrenal insufficiency
**Vanishing white matter disease (VWM)**	Autosomal recessive	*EIF2B1–EIF2B5*	Impaired eIF2B-mediated cellular stress response, resulting in astrocyte and oligodendrocyte vulnerability and progressive white matter rarefaction	Cerebellar ataxia, spasticity, stress-provoked neurologic deterioration (e.g., fever, trauma), developmental regression	No established disease-modifying therapy; supportive care and avoidance of known stressors/triggers; experimental molecular-targeted approaches under investigation
**CSF1R-related disorder (ALSP spectrum)**	Typically, autosomal dominant	*CSF1R*	Primary microglial dysfunction leading to impaired white matter homeostasis, secondary demyelination, and axonal spheroid formation	Rare early-onset presentations have been described and may reflect a broader disease spectrum, though the roles of variant severity and ascertainment remain incompletely defined.	No established disease-modifying therapy; experimental microglia-targeted approaches under investigation
**Alexander disease (astrocytopathy)**	Autosomal dominant in most cases	*GFAP*	Gain-of-function GFAP variants cause astrocytic accumulation of Rosenthal fibers, which are cytoplasmic inclusions composed predominantly of aggregated GFAP together with stress-response proteins such as αB-crystallin and HSP27. Although these aggregates represent abnormal protein deposition, they are not generally regarded as classical amyloid structures, and their precise higher-order structural properties remain incompletely defined.	Developmental delay, macrocephaly (infantile form), seizures, bulbar dysfunction	Supportive therapy; antisense oligonucleotide and other gene-modulating strategies under investigation

**Legend:** * Representative causative genes are shown; additional rare genetic causes or modifiers may exist for some phenotypes. ** Therapeutic availability, regulatory status, and eligibility criteria vary by disease subtype, disease stage, patient age, and jurisdiction. Abbreviations: CNS, central nervous system; GFAP, glial fibrillary acidic protein; HSCT, hematopoietic stem cell transplantation; PNS, peripheral nervous system; VLCFA, very-long-chain fatty acid; VWM, vanishing white matter disease.

**Table 4:** Mitochondrial Dysfunction in Pediatric Neurodegenerative Diseases.

Disorder	Genetic Basis	Core Mitochondrial Defect	Clinical Features
Leigh Syndrome	Mutations in mtDNA/nDNA (e.g., *SURF1*)	ETC dysfunction, impaired oxidative phosphorylation	Psychomotor regression, lactic acidosis
MELAS	mtDNA mutations (e.g., A3243G)	Impaired mitochondrial protein synthesis	Stroke-like episodes, seizures, lactic acidosis
MERRF	mtDNA tRNA mutations	Reduced oxidative phosphorylation	Myoclonus, epilepsy, ataxia
LBSL	*DARS2* gene mutation	Impaired aspartyl-tRNA synthetase, energy deficits	Spasticity, ataxia, leukoencephalopathy
NARP Syndrome	*MT-ATP6* gene mutation	ATP synthase dysfunction	Neuropathy, ataxia, retinitis pigmentosa

Abbreviations legend: **mtDNA** = mitochondrial DNA; **nDNA** = nuclear DNA; **ETC** = electron transport chain; **MELAS** = *Mitochondrial Encephalomyopathy, Lactic Acidosis, and Stroke-like Episodes*; **MERRF** = *Myoclonic Epilepsy with Ragged-Red Fibers*; **LBSL** = *Leukoencephalopathy with Brain Stem and Spinal Cord Involvement and Lactate Elevation*; **NARP** = *Neuropathy, Ataxia, and Retinitis Pigmentosa*; **tRNA** = transfer RNA; **ATP** = adenosine triphosphate; ***MT-ATP6*** = mitochondrial gene encoding subunit 6 of ATP synthase; ***SURF1*** = nuclear gene involved in the assembly of complex IV of the mitochondrial respiratory chain; ***DARS2*** = gene encoding mitochondrial aspartyl-tRNA synthetase.

**Table 5:** Classification and Key Features of Representative Peroxisomal Disorders.

Disorder	Genetic Cause	Pathophysiological Mechanism	Major Clinical Features
**Zellweger spectrum disorders**	Mutations in *PEX* genes	Defective peroxisome assembly and matrix protein import	Hypotonia, seizures, liver dysfunction, craniofacial abnormalities
**X-linked adrenoleukodystrophy**	*ABCD1* mutation	Impaired VLCFA transport into peroxisomes	Demyelination, adrenal insufficiency
**Refsum disease**	*PHYH* or *PEX7* mutation	Defective phytanic acid α-oxidation	Retinitis pigmentosa, neuropathy, ataxia

**Legend:** *ABCD1*—ATP-binding cassette subfamily D member 1; PEX—Peroxin (peroxisomal biogenesis factor); PHYH—Phytanoyl-CoA 2-hydroxylase; VLCFA—Very long-chain fatty acid; ZSD—Zellweger spectrum disorder.

**Table 6:** Diagnostic Tools in Pediatric Neurodegenerative Diseases.

Diagnostic Modality	Indication	Strengths	Limitations
MRI	All suspected cases	Pattern recognition, non-invasive	May be non-specific early
Metabolic screening	Acute/regressive cases	Identifies treatable disorders	Low sensitivity overall
Enzyme assays	Suspected LSDs	High specificity	Limited availability
Gene panels	Clear phenotype	High coverage	Misses novel genes
WES/WGS	Unexplained cases	High diagnostic yield	Variants of uncertain significance
Multi-omics	Unsolved cases	Functional resolution	Research-level availability

**Table 7:** Disease-Specific Precision Therapies in Pediatric Neurodegenerative Diseases.

Disorder	Main Pathogenic Target/Pathway	Therapeutic Modality	Delivery Platform	Representative Therapy	Optimal Treatment Window/Key Timing Principle
**Spinal muscular atrophy (SMA)**	SMN1 deficiency causing motor neuron degeneration	Gene replacement therapy	In vivo AAV9-mediated systemic gene transfer	Onasemnogene abeparvovec	Greatest benefit is achieved before substantial motor neuron loss, ideally in presymptomatic or very early symptomatic infants [139].
**Metachromatic leukodystrophy (MLD)**	ARSA deficiency with sulfatide accumulation and progressive demyelination	Hematopoietic stem cell gene therapy	Ex vivo lentiviral transduction of autologous hematopoietic stem cells, followed by reinfusion	Atidarsagene autotemcel	Best outcomes are observed in presymptomatic or early symptomatic patients, before major neurological decline is established [140].
**Cerebral X-linked adrenoleukodystrophy (cALD)**	ABCD1 defect with VLCFA accumulation, inflammatory demyelination, and cerebral white-matter injury	Hematopoietic stem cell gene therapy	Ex vivo lentiviral correction of autologous CD34+ cells, followed by reinfusion	Lentiviral HSC gene therapy/eli-cel platform	Treatment is most effective at the early cerebral stage, when inflammatory cerebral disease is present but severe neurological disability has not yet developed [141].
**CLN2 disease**	TPP1 deficiency with lysosomal dysfunction and progressive loss of motor-language function	Enzyme replacement therapy	Intracerebroventricular (ICV) enzyme delivery directly targeting the CNS.	Cerliponase alfa	Therapy should begin as early as possible after diagnosis, before advanced functional deterioration, to maximize preservation of motor and language function [142].

**Abbreviations:** SMA, spinal muscular atrophy; SMN1, survival motor neuron 1; SMN2, survival motor neuron 2; AAV9, adeno-associated virus serotype 9; MLD, metachromatic leukodystrophy; ARSA, arylsulfatase A; HSC, hematopoietic stem cell; cALD, cerebral X-linked adrenoleukodystrophy; ABCD1, ATP-binding cassette subfamily D member 1; VLCFA, very-long-chain fatty acids; CLN2, neuronal ceroid lipofuscinosis type 2; TPP1, tripeptidyl peptidase 1; ICV, intracerebroventricular; CNS, central nervous system.

## Data Availability

The data (selected literature) presented in this review article are available on request from the first author.

## Abbreviations

**AAV**	Adeno-associated virus
**AAV9**	Adeno-associated virus serotype 9
**ABCD1**	ATP-binding cassette subfamily D member 1
**ALD**	Adrenoleukodystrophy
**ARSA**	Arylsulfatase A
**ASOs**	Antisense oligonucleotides
**ATP**	Adenosine triphosphate
**cALD**	Cerebral X-linked adrenoleukodystrophy
**CLN1 (PPT1)**	Ceroid lipofuscinosis neuronal 1
**CLN2**	Neuronal ceroid lipofuscinosis type 2
**CNV**	Copy number variation
**CNS**	Central nervous system
**CSF**	Cerebrospinal fluid
**DARS2**	Gene encoding mitochondrial aspartyl-tRNA synthetase
**DHA**	Docosahexaenoic acid
**DNA**	Deoxyribonucleic acid
**ER**	Endoplasmic reticulum
**ERT**	Enzyme replacement therapy
**ES**	Exome sequencing
**ETC**	Electron transport chain
**GAA**	Acid α-glucosidase
**GALC**	Galactocerebrosidase
**GBA1**	Glucosylceramidase beta 1 gene
**GFAP**	Glial fibrillary acidic protein
**GM2**	Monosialotetrahexosylganglioside
**GS**	Genome sequencing
**GWMD**	Genetic white matter disorders
**HSC**	Hematopoietic stem cell
**HSCT**	Hematopoietic stem cell transplantation
**HEXA**	Hexosaminidase subunit alpha gene
**ICD**	International Classification of Diseases
**ICV**	intracerebroventricular
**IDUA**	Alpha-L-iduronidase gene
**LBSL**	Leukoencephalopathy with brain stem and spinal cord involvement and lactate elevation
**LSD**	Lysosomal storage disorder
**LSDs**	Lysosomal storage disorders
**MELAS**	Mitochondrial encephalomyopathy, lactic acidosis, and stroke-like episodes
**MERRF**	Myoclonic epilepsy with ragged-red fibers
**MLD**	Metachromatic leukodystrophy
**MPS I**	Mucopolysaccharidosis type I
**MRI**	Magnetic resonance imaging
**MT-ATP6**	Mitochondrial gene encoding subunit 6 of ATP synthase
**mtDNA**	Mitochondrial DNA
**NARP**	Neuropathy, ataxia, and retinitis pigmentosa
**NCLs**	Neuronal ceroid lipofuscinoses
**NDDs**	Neurodegenerative diseases
**nDNA**	Nuclear DNA
**NGS**	Next-generation sequencing
**NPC1**	Niemann–Pick disease, type C1 gene
**NPC2**	Niemann–Pick disease, type C2 gene
**OMIM**	Online Mendelian Inheritance in Man
**PBD**	Peroxisome biogenesis disorder
**PEX**	Peroxin (peroxisomal biogenesis factor)
**PHYH**	Phytanoyl-CoA 2-hydroxylase
**PIND**	Progressive intellectual and neurological impairment
**PNS**	Peripheral nervous system
**ROS**	Reactive oxygen species
**SMA**	Spinal muscular atrophy
**SMN1**	Survival motor neuron 1
**SMN2**	Survival motor neuron 2
**SMPD1**	Sphingomyelin phosphodiesterase 1 gene
**SRT**	Substrate reduction therapy
**SURF1**	Nuclear gene involved in the assembly of complex IV of the mitochondrial respiratory chain
**TFEB**	Transcription factor EB
**TPP1**	Tripeptidyl peptidase 1
**tRNA**	Transfer RNA
**UPS**	Ubiquitin–proteasome system
**VLCFA**	Very-long-chain fatty acid
**VUS**	Variants of uncertain significance
**VWM**	Vanishing white matter disease
**WES**	Whole-exome sequencing
**WGS**	Whole-genome sequencing
**ZSD**	Zellweger spectrum disorder
**↑**	Increasing
**↓**	Decreasing
